# *Oplr16* serves as a novel chromatin factor to control stem cell fate by modulating pluripotency-specific chromosomal looping and TET2-mediated DNA demethylation

**DOI:** 10.1093/nar/gkaa097

**Published:** 2020-02-14

**Authors:** Lin Jia, Yichen Wang, Cong Wang, Zhonghua Du, Shilin Zhang, Xue Wen, Lei Zhou, Hui Li, Huiling Chen, Dan Li, Songling Zhang, Wei Li, Wei Xu, Andrew R Hoffman, Jiuwei Cui, Ji-Fan Hu

**Affiliations:** 1 Key Laboratory of Organ Regeneration and Transplantation of Ministry of Education, Stem Cell and Cancer Center, First Hospital, Jilin University, Changchun, Jilin 130061, P.R. China; 2 Stanford University Medical School, VA Palo Alto Health Care System, Palo Alto, CA 94304, USA; 3 Department of Endocrinology, Xiangya Hospital, Central South University, Changsha, Hunan, P.R. China

## Abstract

Formation of a pluripotency-specific chromatin network is a critical event in reprogramming somatic cells into pluripotent status. To characterize the regulatory components in this process, we used ‘chromatin RNA *in situ* reverse transcription sequencing’ (CRIST-seq) to profile RNA components that interact with the pluripotency master gene *Oct4*. Using this approach, we identified a novel nuclear lncRNA *Oplr16* that was closely involved in the initiation of reprogramming. *Oplr16* not only interacted with the *Oct4* promoter and regulated its activity, but it was also specifically activated during reprogramming to pluripotency. Active expression of *Oplr16* was required for optimal maintenance of pluripotency in embryonic stem cells. *Oplr16* was also able to enhance reprogramming of fibroblasts into pluripotent cells. RNA reverse transcription-associated trap sequencing (RAT-seq) indicated that *Oplr16* interacted with multiple target genes related to stem cell self-renewal. Of note, *Oplr16* utilized its 3′-fragment to recruit the chromatin factor SMC1 to orchestrate pluripotency-specific intrachromosomal looping. After binding to the *Oct4* promoter, *Oplr16* recruited TET2 to induce DNA demethylation and activate *Oct4* in fibroblasts, leading to enhanced reprogramming. These data suggest that *Oplr16* may act as a pivotal chromatin factor to control stem cell fate by modulating chromatin architecture and DNA demethylation.

## INTRODUCTION

Somatic cells can be reprogrammed by defined factors to achieve plasticity and ultimately to convert into induced pluripotent stem cells (iPSCs) (1). During the initiation of this reprogramming process, 3D chromatin matrixes surrounding key stemness genes must be remodeled to form a specific pluripotency-specific architecture in chromosome territories, topologically associated domains and chromatin loops (2–5). These interactions require juxtaposition of DNAs from different domains within a chromosome or from different chromosomes. Physical intrachromosomal interactions between gene promoters and distal enhancer elements of stemness genes play an important role in the transcriptional regulation of these genes, leading to the initiation of pluripotency (6–8). These promoter-enhancer loops are largely organized by mediator and cohesion protein complexes (9). However, it is not clear how mediator and cohesion protein complexes are guided to stemness gene loci to form the pluripotency-specific intrachromosomal looping. Identification of factors that regulate these 3-dimensional structures might improve the efficiency of the reprogramming process.

In addition to chromatin factors, long noncoding RNAs (lncRNAs) have recently been shown to play a major role in gene regulation (10,11). LncRNA are defined as non-coding transcripts that are longer than 200 nucleotides and transcribed by RNA polymerase II; they are frequently 5′-capped, spliced and polyadenylated (12). Some lncRNAs, such as *Kcvq1ot1*, regulate their target genes by participating in the formation of long distance intrachromosomal loops (13). LncRNAs can regulate the transcriptome of core pluripotency transcription factors (14–18) through epigenetic, transcriptional, and post-transcriptional mechanisms (19). In some cases, lncRNAs may be necessary for the formation of iPSCs from somatic cells (20). It would be important to learn if lncRNAs promote pluripotent reprogramming by controlling the formation of promoter-enhancer chromatin loops in pluripotency-related gene domains.

In this study, we explored the role of lncRNAs in the conversion of fibroblasts into iPSCs. By utilizing a combined ‘chromatin RNA *in situ* reverse transcription sequencing’ (CRIST-seq) (21) and RAT-seq (22) strategy, we identified *Oplr16* (*Oct4*promoter-interacting long noncoding RNA 16) as a novel nuclear-genome derived lncRNA that is associated with somatic cell reprogramming. *Oplr16* epigenetically induces the activation of stem cell core factors by coordinating intrachromosomal looping and recruitment of DNA demethylase TET2. This study highlights the potential role of *Oplr16* in the enhancement of reprogramming for regenerative medicine.

## MATERIALS AND METHODS

### Cell reprogramming

Fibroblasts cultured in six-well plates were infected with *Oct4*-*Sox2*-*Klf4-c-Myc* (OSKM) lentivirus with polybrene (8 μg/ml). Three days later, the cells were collected and transferred on mitomycin C-inactivated MEF feeder cells. The media were replaced with ES medium (DMEM high glucose, 10% FBS, 10% KSR, 1% Glutamax, 1% sodium pyruvate, 1% non-essential amino acids, 0.1% β-mercaptoethanol, 1000 U/ml LIF, 2 μg/ml doxycycline) (23). Both iPSCs and un-reprogrammed cells were collected for further studies (6,24).

### RNA-seq to identify differentially expressed lncRNAs in reprogramming

Total RNA was isolated from fibroblasts and iPSCs (6,25) using TRIzol (Invitrogen, Carlsbad, CA, USA). The indexed libraries were prepared using Illumina's TruSeq RNA Sample Prep Kit v2. Paired-end sequencing was performed by Shanghai Biotechnology (Shanghai, PRC) using a HiSeq4000 (Illumina). RNA-seq yielded 145 million raw reads for iPSC and 148 million raw reads for fibroblasts. After Seqtk filtering, a total of 120 million clean reads for mRNAs and 124 million clean reads of lncRNAs were mapped to the mouse genome (genome version: mm10, GRCm38.p4 (ftp://ftp.ensembl.org/pub/release-83/fasta/mus_musculus/dna/Mus_musculus.GRCm38.dna.primary_assembly.fa.gz) using the STAR software (26). Gene counts were normalized to the values of reads per kilobase of transcript per Million mapped reads (RPKM). Cuffdiff was used to calculate the differentially expressed RNAs when the fold-change was >2 and *P* < 0.05 with an unpaired two-sided *t*-test (22,27).

### Cas9-gRNA guided *Oct4* chromatin immunoprecipitation.

A Cas9-guided chromatin immunoprecipitation assay (CRIST-seq) (21) was modified to identify lncRNAs that bind to the *Oct4* promoter. The Cas9-*Oct4* gRNA vector was constructed by cloning two *Oct4* promoter gRNAs (Supplementary Table S1) into the lentiCRISPR-EGFP sgRNA 2 vector (Addgene Plasmid #51761). iPSCs were transfected with the Cas9-gRNAs lentiviruses. After selection by puromycin, cells were collected for immunoprecipitation (28,29). To assay the *Oct4* promoter-interacting lncRNA, cells were cross-linked and lysed. Nuclei were collected and reverse transcribed with biotin dNTP (10 mM dNTP with 1:20 biotin-dCTP). After nuclear lysis, the chromatin complex was subjected to sonication, and then the biotinylated lnc-cDNA/Cas9 complex was immunoprecipitated with anti FLAG-Cas9 antibody (F1804, Sigma, MO, USA). After cross-link reversal and proteinase K treatment, the biotin-labeled lnc-cDNAs were further purified from genomic DNAs with M-280 streptavidin beads (Invitrogen 11205D). Second strand cDNA was synthesized by using Stratagene cDNA Synthesis kit (Agilent Technologies, CA, USA) for Illumina lnc-cDNA sequencing. The double-stranded cDNAs were digested by MboI (Thermo Scientific FD0814) and ligated with the NEBNext adaptors (NEBNext^®^ ChIP-seq Library Prep Master Mix Set for Illumina). The library DNAs were subject to Illumina sequencing (Shanghai Biotechnology, Shanghai).

We used a random gRNA (gCT) as control and constructed the control library for sequencing using the same protocol. Meanwhile, we used an anti-IgG antibody as the background control for immunoprecipitation (27).

### RNA Extraction and RT-PCR

Total RNA was isolated using TRIzol reagent (Sigma, MO, USA), and then M-MLV (Invitrogen 28025) was used to synthesis cDNA. The RT-PCR reaction was performed with a Bio-Rad Thermol Cycler. All primers utilized are listed in Supplementary Table S1. RT-PCR analyses were performed using FastStart Universal SYBR Green Master (ROX) (Roche 04913914001) on the ABI PRISM 7900HT Sequence Detection System (6,30). Target amplification was performed by PCR of 1 cycle at 95°C for 5 min, 33 cycles at 95°C for 20 s, 62°C for 15 s and 72°C for 15 s and 1 cycle at 72°C for 10 min. The threshold cycle (Ct) values of target genes were assessed and were normalized over the Ct of the β-ACTIN control.

### RNA FISH

The RNA FISH protocol was modified and performed as previously described (31,32). Briefly, the probe for RNA FISH was prepared as antisense single strand DNA (ssDNA) by asymmetric PCR and labeled with digoxygenin. After purification, 0.1 μg ssDNA probe and 10 μg salmon sperm DNA (Boehringer, Meylan, France) were precipitated and suspended in 10 μl RNA hybridization buffer (2× SSC, 10% dextran sulfate, 0.2 mg/ml BSA (Invitrogen, CA, USA), 2 mM VCR, 10% formamide). Hybridization was carried out in a moist chamber at 37°C for 12–16 h. The cells were incubated with anti-digoxygenin (Sigma, 11207741910) for 4 h at room temperature. Cells were washed by PBS three times and incubated in DAPI for 5 min at room temperature. Cells were observed with an inverted fluorescence phase contrast microscope (KEYENCE BZ-X710).

### Embryoid body differentiation

iPSCs were pre-plated to remove feeder cells, and then diluted to 100 000 cells/ml in the medium without LIF. Cells were adherent to the lid of the Petri dish, while the bottom of the dish is filled with PBS, then the lid was reversed and placed over the bottom. Eight dishes were prepared and cells on two dishes were collected every other day and used for gene expression analysis using quantitative PCR.

### Knockdown of *Oplr16* lncRNA

Four shRNAs were cloned into two separate pGreenPuro vectors (#SI505A-1, SBI, CA) to knock down *Oplr16* (Supplementary Table S1). Vector shOplr16-1 contained shRNAs #1 and #3, and vector shOplr16-2 carried shRNAs #2 and #4. The shRNAs were under the control of the H1 and U6 promoters, separately, in each vector. After lentiviral infection, E14 cells and iPSCs were selected by puromycin and tracked by copGFP reporter in the lentiviral vector. Two random shRNAs (5′-GCAGCAACTGGACACGTGATCTTAA-3′ and 5′- TGAAATGTACTGCGCGTGGAGACTA-3′) were cloned in the same vector to construct the assay control (shCT). The copGFP-positive cells were isolated for quantitative PCR.

### The *Oct4* promoter-luciferase assay

A DNA fragment of the *Oct4* promoter and part of exon 1 was amplified by PCR, and cloned into a pGL3 vector. HEK293T cells were transfected with a mixture of p*Oct4*-luciferase plasmid, pRL-TK-renilla-luciferase plasmid and *Oplr16* lentiviral expression plasmid using Lipofectamine 3000 (Invitrogen, CA, USA). The empty lentiviral vector was used as control. After 48 h, cells were collected. Dual-Luciferase Reporter Assay System was used to measure luciferase activity according to the manufacturer's instructions (Promega E1910). All luciferase assays were repeated three times.

### Identification of the *Oplr16* target genes by RNA reverse transcription associated trap sequencing (RAT-seq)

To identify the interacting target genes of *Oplr16*, a RAT-seq assay was modified (33–35). Cells were cross-linked and lysed. Nuclei were suspended and were reverse transcribed with biotin–dNTP. After nuclear lysis, the chromatin complex was subjected to sonication, and the biotinylated lnc-cDNA/chromatin DNA complex was pulled down with M-280 streptavidin beads (Invitrogen 11205D). After cross-linking reversal and proteinase K treatment, genomic DNAs that interact with *Oplr16* were extracted and digested by MboI and ligated with the NEBNext adaptors (NEBNext^®^ ChIP-Seq Library Prep Master Mix Set for Illumina) to construct the library. The library DNAs were subjected to Illumina sequencing (Shanghai Biotechnology, Shanghai) (36). For RAT-seq control, we replaced *Oplr16* complementary primers with random primers to perform a RAT assay, and using the same protocol, constructed a control library (22).

After RAT sequencing, clean reads were mapped to the mouse genome (genome version: mm10) using the Bowtie (version:0.12.8) software (37). MACS2 (version: 2.1.1) was used to identify the enriched regions of the genome by comparing the RAT-seq peaks to input samples; a *q*-value of 0.05 was used as the initial cutoff threshold to minimize peak caller bias (38). To reduce the background, the RAT-seq data were further normalized over the peaks of the control RAT-seq data that were generated by using random oligonucleotide primers in the RAT assay. Differential binding analysis was performed with the DiffBind package using parameters of fold change difference ≥2 and *P*-value < 0.05, with false discovery rate (FDR) <0.1 (22).

### *Oplr16* promotes DOX-OSKM reprogramming

Full length *Oplr16* cDNA was cloned into a pCMV-RsRed-Puro vector, and the construct was confirmed by DNA sequencing. Meanwhile the empty vector (Vector) and vector with random sequence (LncR-CT) were used as controls. The lentiviruses were packaged in H293 cells. OG2 MEFs were transfected with *Oplr16* expression and control lentiviruses and selected by puromycin. MEFs were reprogrammed following the method as previously described (39). Briefly, 20 000 lentivirus-transfected MEFs were seeded in 12-well plates and were cultured in KSR iPSC medium (DMEM high glucose, 10% FBS, 10% KSR, 1% Glutamax, 1% sodium pyruvate, 1% non-essential amino acids, 0.1% β-mercaptoethanol, 1000 U/ml LIF, 2 μg/ml doxycycline). The medium was changed every day. After 10 days, the iPSC colonies were immunostained with Rabbit anti-NANOG Antibody (A300-397A, Bethyl, 1:500 dilution). Positive iPSC colonies per field were recorded (24).

### Immunohistochemical staining of stem cell markers

Fluorescent Mouse ES/iPS Cell Characterization kit (#SCR077, Millipore, MA) was used to identify the pluripotency of stem cells following the protocol provided by the manufacturer. Fluorescence images were acquired with a Zeiss AxioCam Camera.

### Chromosome conformation capture (3C)

As previously reported (40), 1–5 million cells were crosslinked by 2% formaldehyde at room temperature for 10 min, and glycine was then used for quenching. Lysis buffer was used to lyse cells, followed by MboI (NEB, R0147) digestion at 37°C for at least 2 h. T4 DNA ligase (NEB, M0202) was used for ligation. Proteinase K was used to reverse the crosslinks, and DNA was then purified. The primers for 3C PCR were derived from various regions of *Oct4* (5′enhancer, promoter and 3′enhancer). The 3C PCR products were inserted into a pJET Vector (Thermo, K1232) for sequencing. By checking the MboI ligation site, intrachromosomal interactions were confirmed. The 3C ligation products were quantified by using FastStart Universal SYBR Green Master (ROX) (Roche 04913914001) on the ABI PRISM 7900HT Sequence Detection System (41).

### SMC1-lncRNA in situ transcription trap sequencing assay

To assay the SMC1 protein interacting lncRNA, cells were cross-linked and lysed. Nuclei were collected and reverse transcribed with biotin dNTP. After nuclear lysis, the chromatin complex was subjected to sonication, then the biotinylated lnc-cDNA/SMC1 complex was immunoprecipitated with anti-SMC1 antibody (Abcam #ab9262). After cross-linking reversal and proteinase K treatment, DNAs were released. The biotin-labeled lnc-cDNAs were further purified from genomic DNAs with M-280 streptavidin beads (Invitrogen 11205D). The second strand cDNA was synthesized by using Stratagene cDNA Synthesis kit (Agilent Technologies, CA, USA) for Illumina lnc-cDNA sequencing. The double-stranded cDNAs were digested by MboI (Thermo Scientific FD0814) and ligated with the NEBNext adaptors (NEBNext^®^ ChIP-Seq Library Prep Master Mix Set for Illumina). The library DNAs were subjected to Illumina sequencing (Shanghai Biotechnology, Shanghai).

### Validation of the *Oplr16–Oct4* interaction by ChIRP

The *Oplr16–Oct4* interaction was also validated by a ChIRP assay (Chromatin Isolation by RNA Purification) (42). E14 cells were cross-linked, lysed and sonicated. Three 3′-end biotinylated oligonucleotide DNA probes were hybridized to the *Oplr16* lncRNA. The biotinylated probe/*Oplr16*/chromatin DNA complex was pulled down with streptavidin C1 beads (Invitrogen 65002). The *Oplr16-*pulldown chromatin DNA was extracted and quantitated by qPCR for the interacted *Oct4*.

### RNA pull-down

RNAs were transcribed by using T7 RNA polymerase (NEB M0251) and labeled with biotin-CTP. Biotinylated RNAs were incubated with iPSC nuclear extracts, then streptavidin agarose beads (Invitrogen 11205D) were added to each binding reaction and further incubated at RT. Beads were washed and boiled in SDS buffer. Then the retrieved proteins were detected by Western blot.

### DNA methylation analysis in the gene promoter

Genomic DNAs were extracted from untreated and treated fibroblasts. EZ DNA Methylation-Gold Kit (Zymo D5005) was used to methylate DNAs. Primers for methylation PCR are listed in Supplementary Table S1. 2× BlueStar (Mclab, BSPM-200) was used for methylation PCR. The amplification process was: 1 cycle at 95°C for 10 min, 32 cycles at 95°C for 20 s, 62°C for 15 s and 72°C for 15 s and 1 cycle at 72°C for 5 min. The PCR products were purified and cloned into pJET vector for sequencing as previously described. After bisulfite treatment, unmethylated cytosines were converted to uracils, while the methylated cytosines remain as cytosines (43,44).

### Statistical analysis

The experimental data were prepared and expressed as mean ± SD. Data were analyzed using SPSS software (version 16.0; SPSS, Inc., IL, USA). Student's *t*-test or one-way ANOVA (Bonferroni test) was performed to compare statistical differences among treatment groups. For every sample, two sets of duplicates were averaged for each of three biological replicates to obtain a final *n* of three for all statistical analyses. Results were considered statistically significant at *P* < 0.05. Error bars represent the standard deviation.

## RESULTS

### Identification of *Oplr16* as a pluripotent lncRNA by CRIST-seq

**Figure 1.**
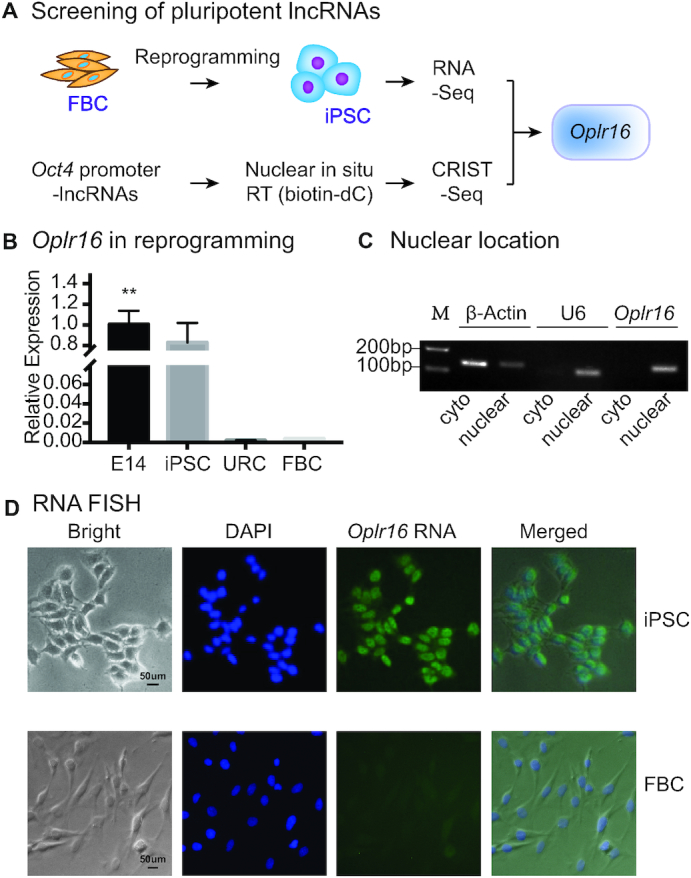
Identification of *Oplr16* as a pluripotent lncRNA. (**A**) Identification of *Oplr16* as a pluripotency-associated lncRNA by integrating RNA-seq and CRIST-seq. RNA-seq was used to identify RNAs that are differentially expressed between fibroblasts and iPSCs collected in the process of reprogramming. In CRIST-seq, the *Oct4* promoter was targeted by the expression of the Cas9 *Oct4*-gRNA. LncRNAs interacting with *Oct4* promoter were reverse transcribed *in situ* into cDNA with biotin-dCTP. After immunoprecipitation, biotin-cDNAs were purified by streptavidin beads for Illumina sequencing. Both sets of databases were integrated to identify the pluripotency-associated lncRNA *Oplr16*. (**B**) Reactivation of *Oplr16* in reprogramming. Fibroblasts were transfected with a *Oct4-Sox2-Kilf4-c-Myc* (OSKM) lentivirus. Cells were collected at various stages of reprogramming and expression of *Oplr16* was measured by qPCR. E14: mouse embryonic pluripotent stem cell line used as a positive control; iPSC: induced pluripotent stem cells; non-iPSC: un-reprogrammed cells that express four OSKM factors, but fail to complete reprogramming; FBC: fibroblasts. β-Actin was used as the PCR control. ** *P* < 0.01 as compared with FBC and URC. (**C**) *Oplr16* is predominantly located in the nucleus. Cytoplasmic (cyto) and nuclear RNAs were isolated and reverse transcribed into cDNAs. Location of *Oplr16* was determined by PCR. β-Actin: cytoplasm control. U6: nuclear control. (**D**) Confirmation of nuclear location of *Oplr16* by RNA-FISH. Digoxygenin labeled *Oplr16* probe was prepared and hybridized in iPSCs and fibroblasts (FBC). DAPI: nuclear control.

A potential pluripotency-associated lncRNA should meet two criteria. First, it should have the ability to regulate stem cell core factor genes, such as *Oct4*, *Sox2* and *Nanog*. Second, this lncRNA should be differentially expressed in response to the status of pluripotency. Therefore, we proposed to adopt a novel strategy to identify potential lncRNAs that meet these two criteria (Figure 1A). We used a chromatin RNA *in situ* reverse transcription sequencing (CRIST-seq) approach to determine which lncRNAs interact with the *Oct4* promoter (21), a key transcription factor required for reprogramming somatic cells into iPSCs. Then, conventional RNA transcriptome sequencing (RNA-seq) was used to identify differentially-expressed lncRNAs that may be associated with pluripotent reprogramming (22).

Using this strategy, we discovered *Oplr16* (NONMMUT101010) as a novel lncRNA that meets the above criteria. First, *Oplr16* was highly expressed in iPSCs and E14 embryonic stem cells, but there was no expression in fibroblasts and non-iPSCs (Figure 1B, Supplementary Figure S1A). We isolated RNAs from various tissues, including artery, brain, cerebellum, fat, kidney, liver, lung, spleen and heart and found specific expression of *Oplr16* only in E14 embryonic stem cells (Supplementary Figure S1B). Second, the CRIST-seq approach showed that *Oplr16* specifically interacted with the *Oct4* promoter (Supplementary Figure S2). No interaction signal of *Oplr16* was detected in the IgG and random control genes.

We also validated this *Oplr16*-*Oct4* interaction using the previously reported ChIRP (Chromatin Isolation by RNA Purification) (Supplementary Figure S3A) assay (42). LncRNA-chromatin complexes were cross-linked *in vivo*, sonicated, and hybridized with lncRNA-specific biotinylated antisense oligonucleotide probes. After washing, chromatin complexes that interact with the lncRNA were pulled down using magnetic streptavidin beads for qPCR quantitation. Using this approach, we detected an enriched *Oplr16*–*Oct4* interaction signal in the *Oplr16*-specifc biotinylated probe group (Supplementary Figure S3B). No interaction signals were detected in the random probe group or in the control group.

*Oplr16* is a 629 bp lncRNA gene located in the olfactory receptor family gene cluster on mouse chromosome 17 (Supplementary Figure S4). By alignment, an *Oplr16* homolog was located at chromosome 6 in rat (Supplementary Figure S5). However, no homologous sequence was matched in the human genome. These data suggest that *Oplr16* is a pluripotency-associated lncRNA that is specifically transcribed in mouse pluripotent stem cells, but it is not conserved in the human genome.

In a subcellular fractionation assay, we isolated cytoplasmic and nuclear RNAs. RT-PCR showed that *Oplr16* was predominantly located in the nucleus (Figure 1C). An RNA-FISH assay also validated its nuclear localization in iPSCs (Figure 1D). Together, these data suggest that *Oplr16* is a mouse nuclear lncRNA that is activated during somatic reprogramming.

### *Oplr16* is required for the maintenance of stem cell pluripotency

**Figure 2.**
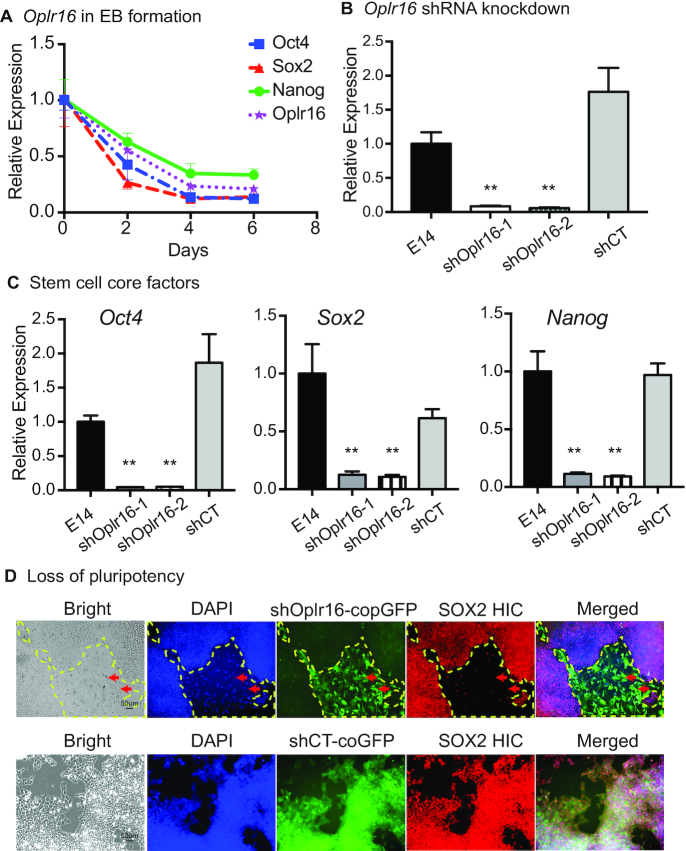
*Oplr16* is required for the maintenance of stem cell pluripotency. (**A**) Expression of *Oplr16* decreased during embryoid body differentiation. iPSCs were collected at Days 2, 4, 6 after EB formation and qPCR was used to quantitate the expression of *Oplr16* in relation to that of stem cell core factor genes *Oct4, Sox2* and *Nanog*. (**B**) Knockdown of *Oplr16* by shRNAs in E14 cells. shOplr16-1 and shOplr16-2: shRNAs that target *Oplr16*; shCT: random shRNA control. ** *P* < 0.01 as compared with iPSC and shCT. (**C**) Downregulation of stem cell core factor genes *Oct4*, *Sox2* and *Nanog* by *Oplr16* knockdown. After transfection with shOplr16-1 and shOplr16-2 lentiviruses, cells were collected for quantitative PCR. ** *P* < 0.01 as compared with iPSC and shCT. (**D**) Knockdown of *Oplr16* induces loss of pluripotency. *Oplr16* was knocked down by shOplr16 lentiviruses in E14 cells. The shCT lentiviruses were used as control. Lentivirus-transfected cells were tracked by copGFP (green). The pluripotency was examined by histoimmunochemical (HIC) staining of stem cell marker SOX2 (red). ShOplr16-transfected E14 cells showed the loss of pluripotent marker SOX2. Cell morphology was also changed. As the control, the shCT treatment did not alter stem cell pluripotency.

To examine the role of *Oplr16* in pluripotency, we collected cells at different days during embryoid body differentiation. Using qPCR, we showed that *Oplr16* was positively associated with the status of pluripotency, sharing a similar expression pattern with *Oct4*, *Sox2* and *Nanog* during embryoid body differentiation (Figure 2A).

We then studied the function of *Oplr16* by knocking down its expression using two separate shRNA lentiviruses (shOplr16-1, shOplr16-2). Each lentiviral vector carried two shRNAs that target *Oplr16* (Supplementary Table S1). E14 cells were transfected with shOplr16 lentiviruses. Lentiviruses carrying random shRNAs were used as the control (shCT). After puromycin selection, we collected the copGFP-positive cells for quantitative PCR. Treatment with both lentiviral shRNAs knocked down *Oplr16* equally in E14 cells (Figure 2B), leading to a significant downregulation of the three stem cell core factors *Oct4*, *Sox2* and *Nanog* (Figure 2C).

After *Oplr16* knockdown, we examined the pluripotency of transfected cells by histoimmunochemical (HIC) staining of stem cell marker SOX2 (red). We found that the shOplr16-1 transfected cells lost the pluripotent marker SOX2 (Figure 2D, top panels 3–4, copGFP-positive cells). We also observed a change in morphology in the shOplr16-1 transfected cells. The random control shCT-transfected cells did not undergo a change in cellular morphology. After *Oplr16* knockdown, E14 cells became very fragile, and they could not proliferate after passage. The cell senescence pathway was activated in *Oplr16* knockdown cells (Supplementary Figure S6). Notably, *Oplr16*-knockdown cells lost the ability of self-renewal, and they failed to form teratomas in nude mice. These data confirmed the requirement of *Oplr16* for the maintenance of pluripotency in E14 cells.

### *Oplr16* promotes pluripotent reprogramming

We then performed a series of assays to examine the role of increased expression of *Oplr16* in stem cells. Using a luciferase assay, we amplified the DNA fragment containing the *Oct4* promoter and a part of exon 1, and cloned it into a pGL3 vector to construct a pOct4-luciferase plasmid (Supplementary Figure S7A). The full length *Oplr16* was also cloned into a pCMV-RsRed-Puro vector. An empty vector and a lncRNA control were used as the assay controls. HEK293T cells were co-transfected with a mixture of the pOct4-luciferase plasmid, the pRL-TK-renilla-luciferase plasmid, and the *Oplr16* expression plasmid or control plasmid DNAs. The luciferase reporter assay demonstrated that *Oplr16* activated the *Oct4* promoter (Supplementary Figure S7B). Future studies are needed to examine the potential mechanisms for the activation in this *in vitro* system that lacks the ability to form intrachromosomal looping, like the R-loop formation and the recruitment of other transcription factors.

**Figure 3.**
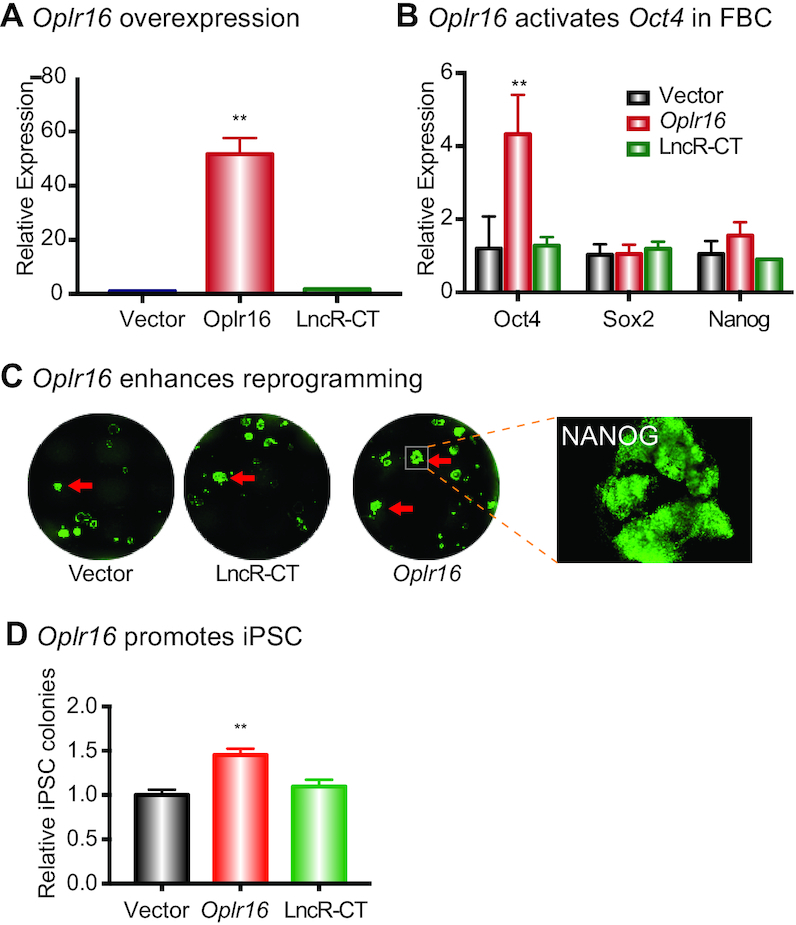
*Oplr16* promotes Reprogramming. (**A**) Overexpression of *Oplr16* in fibroblasts. Fibroblasts were transfected by *Oplr16* lentiviruses and control lentiviruses. After selection by puromycin, stable cells were collected for qPCR quantitation. (**B**) *Oplr16* activated the endogenous *Oct4* in transfected fibroblasts. However, the expression of *Sox2* and *Nanog* show no significant change as compared with Vector and LncR-CT controls. (**C**) *Oplr16* enhanced reprogramming. DOX-inducible MEF cells were transfected with lentiviruses carrying the empty vector, random lncRNA control, and *Oplr16*. After 10 days of doxycycline (DOX) induction, iPSC colonies were immunostained using an antibody against pluripotency maker NANOG (green). (**D**) *Oplr16* promoted iPSC formation. After DOX induction, the number of NANOG positive iPSC colonies was quantitated. ** *P* < 0.01 as compared with the controls.

Then, we examined if *Oplr16* was able to activate endogenous stem cells core factors in fibroblasts, a critical step needed to initiate pluripotent reprogramming. After *Oplr16* lentiviral infection and puromycin selection, fibroblasts were collected for quantitative PCR. *Oplr16* was highly expressed in the transfected fibroblasts (Figure 3A) as was *Oct4*, while the expression of *Sox2* and *Nanog* showed no significant change (Figure 3B).

The role of *Oplr16* in pluripotency was further examined using a DOX-inducible reprogramming system (Supplementary Figure S8). The *Oplr16* lentiviruses were transfected into OG2 MEF cells that carry DOX-inducible OSKM genes. After puromycin selection, doxycycline (DOX) was added to initiate pluripotent reprogramming. After 10 days, iPSC colonies started to form in the *Oplr16*-expressed group (Supplementary Figure S8C). Using immunostaining for the pluripotency marker NANOG (green), we observed more iPSC colonies in *Oplr16*-transfected cells than that in control cells (Figure 3C and D). Cell senescence is a critical barrier to cell reprogramming (45). In the *Oplr16*-treated group, we noted the downregulation of senescence marker genes (Supplementary Figure S9), in parallel with the enhancement of reprogramming.

We further examined the function of *Oplr16* in a ‘rescue’ assay in the LIF-withdrawal cells. E14 cells were transfected with *Oplr16* lentiviruses and seeded at very low density in a medium that lacks LIF. After LIF withdrawal, E14 cells lost pluripotency and became differentiated at very low density. We showed that *Oplr16* partially rescued the defects caused by LIF-withdrawal, including the stem cell spheroid formation and the downregulation of stem cell core factor genes (Supplementary Figure S10).

### *Oplr16* specifically binds to the *Oct4* promoter

**Figure 4.**
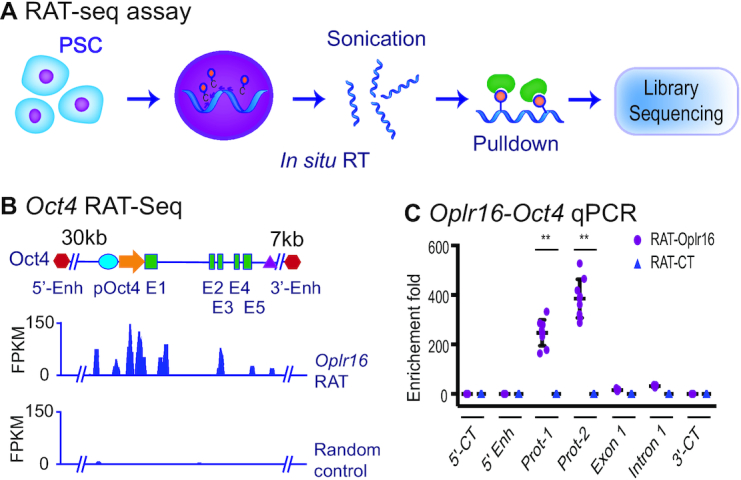
*Oplr16* binds to the *Oct4* promoter. (**A**) Schematic diagram of the RNA reverse transcription-associated trap sequencing (RAT-seq) assay. *Oplr16* lncRNA was *in situ* reverse transcribed using three *Oplr16*-specific complementary primers with biotin-dCTP. The random primers were used as the negative control (RAT-CT). After nuclear lysis, the bitoin-*Oplr16* cDNA chromatin complex was isolated by streptavidin beads and the *Oplr16*-interacting target DNAs were isolated for Illumina library sequencing. (**B**) The IGV analysis of *Oplr16* binding signals in the *Oct4* locus. 5′-Enh, 3′-Enh: the *Oct4* 5′- and 3′-enhancers; pOct4: *Oct4* promoter; E1-E5: *Oct4* exons. (**C**) Quantitative PCR mapping of *Oplr16* binding in the *Oct4* locus. The RAT pulldown complex was used to map the *Oplr16* binding. 5′-CT, 3′-CT: the RAT control sites in the *Oct4* locus. Note the enrichment of the *Oplr16* binding signals in the promoter region (Prot-1 and Prot-2) (*N* = 9, ** *P* < 0.001 as compared with the RAT control).

We then used a RNA reverse transcription-associated trap sequencing (RAT-seq) approach (38) to map the genome-wide targets for *Oplr16* (Figure 4A). E14 cells were collected and *Oplr16* lncRNAs was labeled *in situ* with biotin-dCTP using stringent reverse transcription with three *Oplr16*-specific complementary primers. Random primers were used as the assay control (RAT-CT). The biotin–*Oplr16* cDNA chromatin complex was isolated by streptavidin beads for Illumina library sequencing.

IGV analysis showed enrichment of *Oplr16* at the *Oct4* locus, with a peak in the promoter area. The control RAT showed no binding peaks at that locus (Figure 4B). Quantitative PCR also showed enrichment of *Oplr16* in the *Oct4* promoter area as compared to the RAT-CT control (Figure 4C).

We also examined the binding to another core stem cell factor *Sox2*, which was not activated by *Oplr16* (Supplementary Figures S11A and B). The *Oplr16* RAT IGV analysis showed no peak at the *Sox2* promoter (Supplementary Figures S11C-S11D). Gene ontology (GO) and Kyoto Encyclopedia of Genes and Genomes (KEGG) analyses revealed that *Oplr16* is involved in many signal pathways that are related to development and cell differentiation (Supplementary Figures S12-S13). The top listed target genes were associated with embryonic development and several signal pathways (Supplementary Figure S14).

### *Oplr16* plays an important role in the chromatin looping

**Figure 5.**
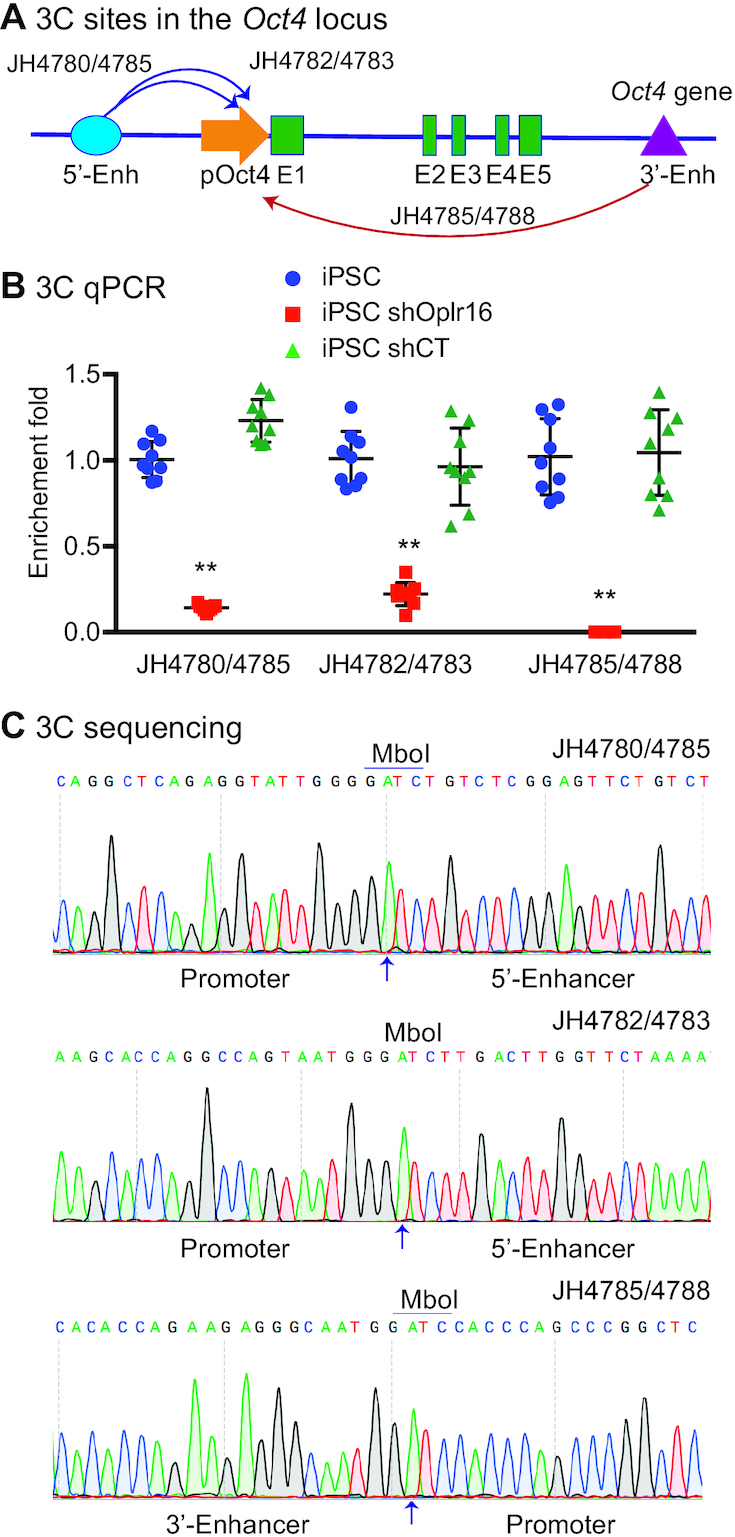
*Oplr16* is essential for the maintenance of intrachromosomal looping in the *Oct4* locus. (**A**) The PCR sites in the *Oct4* locus. Intrachromosomal interactions were quantitated by 3C (chromatin conformation capture) qPCR. 5′-Enh: 5′-enhancers; pOct4: *Oct4* promoter; E1-E5: Exons; 3′-Enh: 3′-Enhancer. Arrows: intrachromosomal interactions. (**B**) Knockdown of *Oplr16* abolished intrachromosomal interaction loops. The 3C interaction was detected by qPCR. For comparison, the relative 3C interaction was calculated by setting the 5′- and 3′- controls as 1 (*N* = 9, ** *P* < 0.001 as compared with the shCT and iPSC controls). (**C**) Sequencing of the *Oct4* intrachromosomal loop products. Arrows: the 3C ligation product containing the MboI site that is flanked by the promoter and the enhancer sequences.

We previously discovered a promoter-enhancer intrachromosomal loop that is critical for the initiation and maintenance of stem cell pluripotency (6). We used a chromosome conformation capture (3C) approach (36,41) to examine if *Oplr16* is involved in this intrachromosomal looping. E14 cells were crosslinked and lysed, followed by MboI digestion and T4 DNA ligation. After proteinase K treatment, DNA was purified for 3C PCR using primers from different regions of *Oct4*, including the 5′-enhancer, promoter and 3′-enhancer (Figure 5A). As previously reported (6), we detected pluripotency-associated 3C loops between the enhancers and promoters in E14 cells. However, knockdown of *Oplr16* abolished these intrachromosomal loops in treated cells (Figure 5B), while the treatment of E14 cells with the shRNA control (shCT) did not interfere with intrachromosomal looping.

We validated these intrachromosomal loops by DNA sequencing of 3C PCR products. As expected, we observed that the MboI ligation site was flanked by the sequences of the *Oct4* promoter and enhancers (Figure 5C). These data suggest that *Oplr16* is important for the formation and/or maintenance of these specific intrachromosomal interactions. Knockdown of *Oplr16* abolished the 3D chromatin structures required for the maintenance of pluripotency.

### *Oplr16* recruits SMC1 to maintain chromatin looping

**Figure 6.**
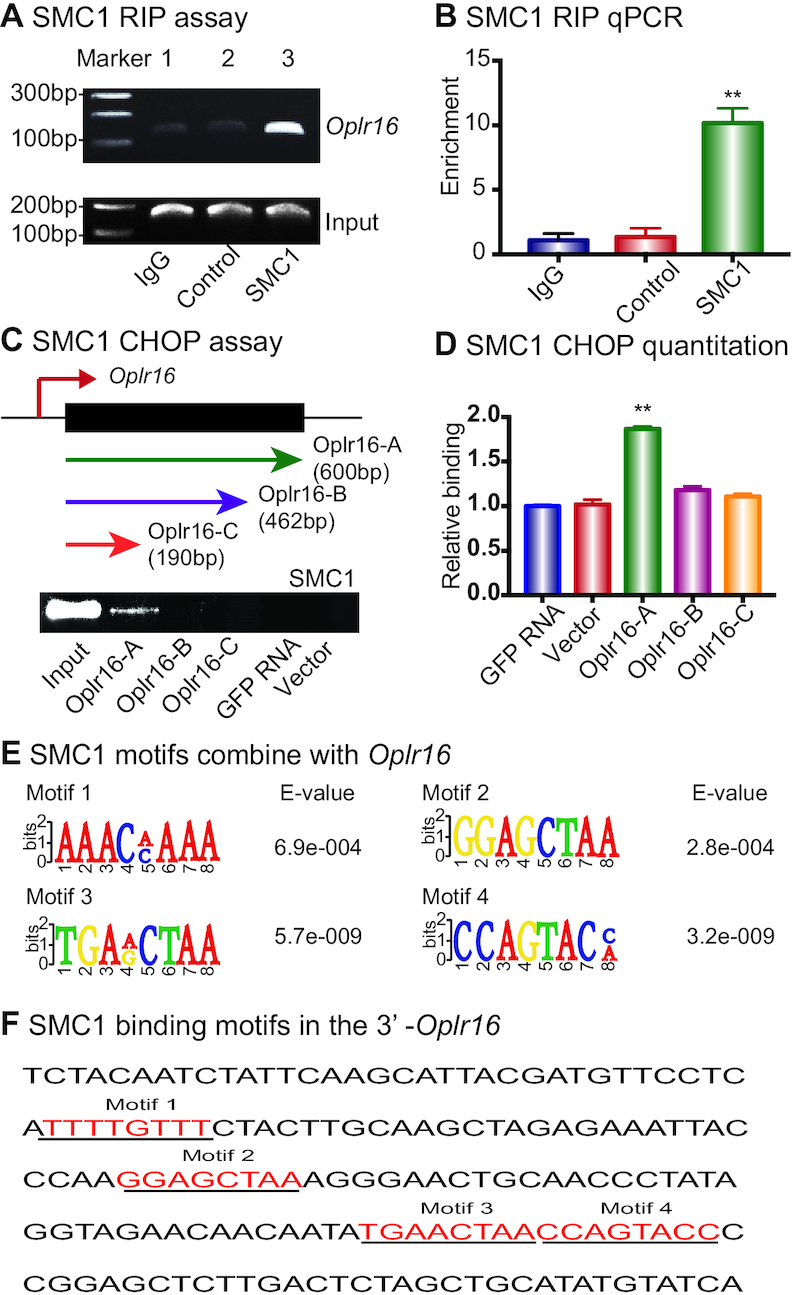
*Oplr16* interacts with chromatin factor SMC1. (**A**) Interaction of *Oplr16* with SMC1 by RNA-chromatin immunoprecipitation (RIP). The SMC1–lncRNA chromatin complex was immunoprecipitated with an antibody against SMC1. The immunoprecipitated RNAs were reverse transcribed and the SMC1-interacting *Oplr16* was measured by PCR. IgG was used as the antibody control. Input: aliquot DNAs collected during the RIP assay. (**B**) Quantitative RIP PCR of the *Oplr16*-SMC1 interaction. (**C**) Identification of the SMC1 binding fragment in *Oplr16*. The chromatin oligo affinity precipitation (CHOP) assay was used to determine the specific fragment of *Oplr16* that interacts with SMC1. Top panel: Schematic diagram of CHOP mapping. Three different sizes of biotin-labeled *Oplr16* fragments were synthesized and bound to streptavidin agarose beads. Recombinant SMC1 proteins were incubated with biotin-*Oplr16* streptavidin beads. After elution, the *Oplr16*-interacted SMC1 proteins were analyzed by Western immunoblotting. Only the full-length *Oplr16* showed the interaction with SMC1 (Oplr16-A). GFP RNA was used as the negative control. Input: SMC1 protein. (**D**) Quantitation of the interaction of SMC1 with *Oplr16* fragments. (**E**) Consensus RNA binding motifs of SMC1 by RIP-seq. (**F**) The presence of SMC1 motifs in the *Oplr16* 3′-fragment.

The cohesion-complex gene SMC1 is critical for intrachromosomal interactions (6). In order to determine if *Oplr16* maintains intrachromosomal looping through SMC1, we used an SMC1 RNA-chromatin immunoprecipitation (RIP) assay and showed that *Oplr16* interacted with SMC1 (Figure 6A and B).

We then used an SMC1 CHOP assay to identify the *Oplr16* fragment that binds to SMC1. Three different sizes of biotin-labeled *Oplr16* were synthesized, purified by streptavidin agarose beads, and incubated with SMC1 recombinant proteins (Figure 6C). After washing, the *Oplr16*-interacting SMC1 was eluted and analyzed by immunoblotting. Full-length *Oplr16* bound to SMC1 (Figure 6D), while the two fragments that lack the 3′-end did not interact with SMC1. We also performed SMC1 RIP sequencing to identify the conserved RNA binding motifs (Figure 6E). Analysis of lncRNA Oplr16 showed that its 3′-fragment contained at least four SMC1 binding motifs (Figure 6F). Collectively, these data suggest that the 3′-fragment of *Oplr16* binds with SMC1.

### *Oplr16* induces DNA demethylation in fibroblasts

**Figure 7.**
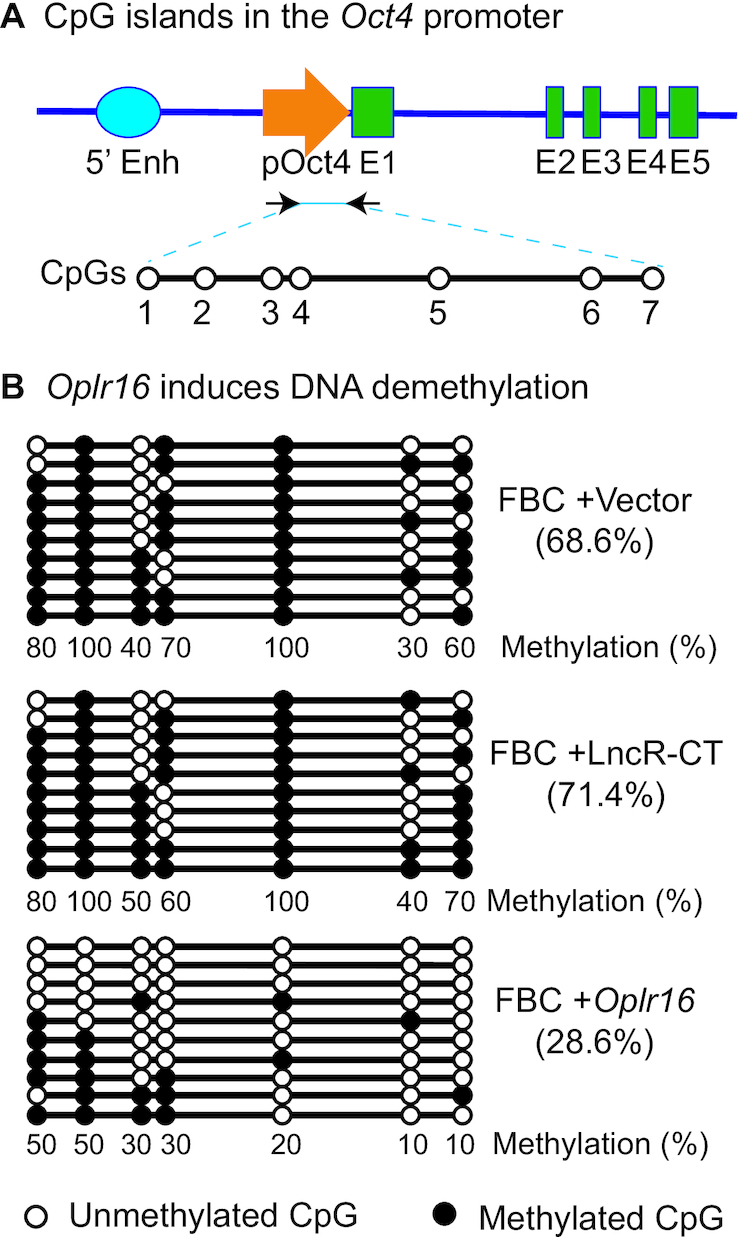
*Oplr16* induces DNA demethylation in fibroblasts. (**A**) CpG islands in the *Oct4* promoter. 5′-Enh: 5′-enhancers; p*Oct4*: *Oct4* promoter; E1–E5: exons. (**B**) *Oplr16* induces DNA demethylation. Fibroblasts (FBC) were transfected with control vector, lncRNA control (LncR-CT) and *Oplr16*. Cells were collected for measurement of DNA methylation by sodium bisulfite sequencing. Solid dot: methylated CpG islands; open dot: unmethylated CpG islands. Numbers under each CpG site: the percentage of methylated CpGs for the CpG site. Numbers in the parenthesis: the percentage of total methylated CpGs over the CpGs in the sequencing. Each line represents the sequence for one clone. A total of 10 clones were sequenced for each group.

Successful initiation of pluripotent reprogramming requires CpG DNA demethylation in stem cell core factor genes, like *Oct4*. To examine if *Oplr16* activates *Oct4* by inducing promoter DNA demethylation, we collected *Oplr16* lentivirus-transfected fibroblasts, extracted genomic DNA, and used a EZ DNA Methylation-Gold Kit to map DNA methylation in the *Oct4* promoter (Figure 7A). Ectopic expression of *Oplr16* induced extensive CpG DNA demethylation in transfected fibroblasts, particularly at the CpG 5 site (Figure 7B). These data suggest that *Oplr16* may promote reprogramming by inducing DNA demethylation in the *Oct4* promoter.

### *Oplr16* recruits TET2 to promote demethylation

**Figure 8.**
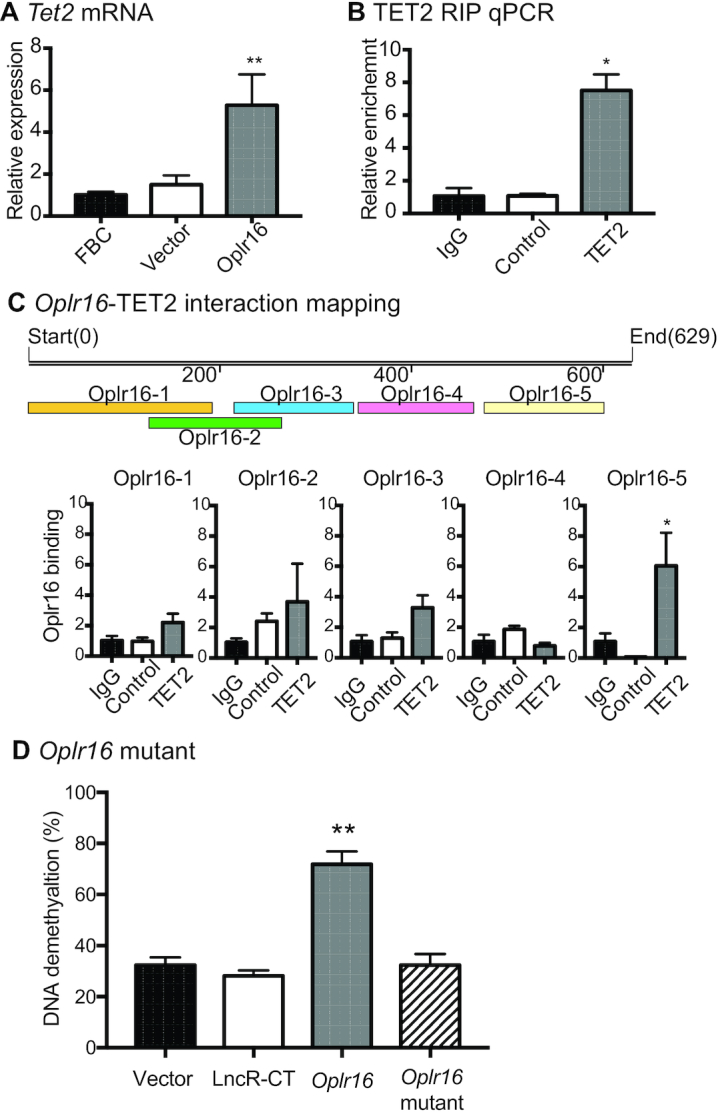
*Oplr16* recruits TET2 to induce DNA demethylation. (**A**) After lentivirus transfection, fibroblasts were collected and quantitative PCR was used to determine the expression of the *Tet2* gene. In *Oplr16* transfected fibroblasts, *Tet2* was upregulated compared to untreated fibroblasts and control vector transfected fibroblasts. (**B**) Interaction of *Oplr16* with TET2 enzyme by RNA-chromatin immunoprecipitation (RIP). The TET2-*Oplr16* lncRNA chromatin complex was immunoprecipitated with an antibody against TET2. After de-crosslinking, the immunoprecipitated RNAs were reverse transcribed. The TET2-interacting *Oplr16* was measured by quantitative PCR. IgG was used as the antibody control. Input: aliquot DNAs collected during the RIP assay. (**C**) Identification of the TET2 binding fragment by RIP mapping. After immunoprecipitation with a TET2 antibody, the TET2-interacting *Oplr16* lncRNA fragments were mapped by quantitative PCR using overlapping primers (color panels). The *Oplr16*-5 (3′fragment) showed strong binding to TET2. For comparison, the value of the IgG control was set as 1. Bottom panel: Schematic diagram of RIP mapping. (**D**) Deletion of the 3′-fragment abolishes the ability of *Oplr16* to induce DNA demethylation. Fibroblasts were transfected with lentiviruses carrying the *Oplr16* mutant that lack the 3′-fragment. After puromycin selection, cells were collected for detecting DNA methylation status. Compared with the full length *Oplr16*, the 3′-fragment-deleted mutant failed to induce DNA demethylation in the *Oct4* promoter. * *P* < 0.05, ** *P* < 0.01 as compared with controls.

We further explored the mechanism by which *Oplr16* initiates DNA demethylation. We were interested to know if the virally-expressed *Oplr16* altered the abundance of the DNA demethylase family of genes *Tet1*, *Tet2* and *Tet3*, and DNA methyltransferase *Dnmt1*. Overexpression of *Oplr16* upregulated *Tet2* in transfected cells (Figure 8A) but had no effect on *Tet1*, *Tet3* and *Dnmt1* (Supplementary Figure S15).

We then used RNA immunoprecipitation (RIP) assay to determine if *Oplr16* interacts with DNA demethylase TET2. The TET2-RNA chromatin complex was immunoprecipitated with an antibody against TET2 and the anti-TET2 immunoprecipitated RNAs were reverse transcribed to quantitate the TET2-interacting lncRNA. There was a significant interaction between *Oplr16* and TET2 as compared with the anti-IgG and lncRNA controls (Figure 8B).

We then mapped the specific fragment that interacts with TET2 using overlapping primers covering the *Oplr16* lncRNA. We found the 3′-fragment of *Oplr16* (*Oplr16*-5) showed the highest interaction with TET2 (Figure 8C). Further mapping showed that all the 3′ sub-fragments had similar binding capacity with TET2 (Supplementary Figure S16).

Finally, we studied the function of *Oplr16* by deleting its 3′-fragment. The *Oplr16* mutant was compared with the wild type *Oplr16* for its ability to induce DNA methylation in the *Oct4* promoter. Fibroblasts were transfected with lentiviruses carrying the mutant and wild type *Oplr16*. After puromycin selection, we examined the status of DNA methylation in transfected cells and found that deletion of the 3′-fragment abolished its ability to induce demethylation in the *Oct4* promoter (Figure 8D, Supplementary Figure S17). These data suggest that after binding to the *Oct4* promoter, *Oplr16* may use its 3′-fragment to recruit TET2 and induce DNA demethylation for the initiation of pluripotent reprogramming. The overlapping binding of TET2, SMC1, *Oplr16*, and histone epigenetic modifications at the *Oct4* promoter is shown in Supplementary Figure S18.

## DISCUSSION

Despite our success in reprograming somatic cells into a pluripotent state, key questions remain unanswered regarding the underlying molecular mechanisms and key factors that control this plasticity switch. In this study, we identified a novel lncRNA, *Oplr16*, that functions as a pivotal factor to regulate pluripotent reprogramming. *Oplr16* is a critical chromatin component of the *Oct4* promoter complex. By binding to the *Oct4* locus, it helps maintain a pluripotency-specific enhancer-promoter intrachromosomal loop and induces DNA demethylation in the gene promoter. Through these two epigenetic mechanisms, *Oplr16* induces the activation of *Oct4*, a core stem cell factor gene, as an essential step to initiate pluripotent reprogramming. Knockdown of *Oplr16* interrupts the intrachromosomal looping and induces the loss of pluripotency in E14 cells. *Oplr16* is closely associated with the status of pluripotency. *Oplr16* is specifically expressed in pluripotent stem cells, including iPSCs and E14 embryonic stem cells. It is not transcribed in fibroblasts and differentiated cells, nor is it expressed in any adult tissues examined so far. However, the *Oplr16* gene switches on during the reprogramming progress in response to pluripotent inducers. Its expression status parallels that of core stem cell factor genes *Oct4*, *Sox2*, and *Nanog* during the process of reprogramming or embryoid body differentiation. Ectopic expression of *Oplr16* enhances the efficiency of fibroblast reprogramming into iPSCs. Collectively, this study demonstrates that *Oplr16* is a unique chromatin lncRNA factor that plays a pivotal role in coordinating a pluripotency-associated topological network and epigenotype that are necessary to initiate reprogramming.

A critical event in the initiation of pluripotent reprogramming is the removal of DNA methylation to activate pluripotency-regulated genes (46–48). However, the mechanisms by which DNA demethylation is initiated specifically in these gene promoters during reprogramming are not fully understood. In this study, we provide the first evidence that *Oplr16* may function as an epigenetic messenger to help induce DNA demethylation in the *Oct4* promoter. *Oplr16* does not encode any chromatin factors. However, we show that ectopic expression of this lncRNA in fibroblasts induces upregulation of *Tet2*, one of the key DNA demethylase family members that are required for the initiation of reprogramming (49). Additionally, lncRNA *Oplr16* binds to the *Oct4* promoter, where it recruits DNA demethylase TET2 and induces DNA demethylation, initiating gene transcription.

We provide further evidence that suggests the critical role of the 3′-fragment of *Oplr16* in DNA demethylation. The 3′-fragment of *Oplr16* contains four consensus SMC1-binding motifs. Genetic deletion of the 3′-fragment abolishes the ability of *Oplr16* to induce DNA demethylation in the *Oct4* promoter. Thus, *Oplr16* may act as a special lncRNA molecule that binds to the Oct4 promoter and guides *de novo* DNA demethylation to activate the gene for reprogramming. However, we cannot exclude the possibility that other epigenotype writers, like *Dnmt1*, *Tet1* and *Tet3*, are also involved in the regulation of *Oct4* and whether the *Oplr16* 3′-end has the ability to bind both SMC1 and TET2 in *in vitro* assays. Similarly, it is still unknown how *Oplr16*-SMC1-TET1 coordinate with each other in chromosomal looping and DNA demethylation.

Another important finding of this study is that *Oplr16* acts like a chromatin factor that helps the formation of pluripotency-associated intrachromosomal looping. Chromatin looping between core promoter elements and distal enhancer elements controls the transcriptional activity of genes (50–52). At the *Oct4* gene locus, for example, the intrachromosomal loops between the gene promoter and enhancer regulatory regions are specific for pluripotent stem cells. This topological structure helps bring distal regulatory elements, like enhancers, into physical proximity with gene target promoters, thereby activating them to initiate cellular reprogramming (53). In this study, we show that *Oplr16* is enriched in the *Oct4* promoter and is essential for the maintenance of this intrachromosomal looping. In E14 embryonic stem cells, knockdown of *Oplr16* abolishes this intrachromosomal long-range interaction, in parallel with the exit of E14 cells from pluripotency.

It is known that Mediator and Cohesin physically and functionally connect enhancers and core promoters of active genes in embryonic stem cells (54). Mediator, a transcriptional coactivator, forms a complex with Cohesin, which can form rings that connect two DNA segments (8,55). We show that by binding to the *Oct4* promoter, *Oplr16* may recruit SMC1 and help orchestrate the promoter-enhancer loops. *Oplr16* is actively transcribed during reprogramming and acts in concert with other chromatin factors to coordinate a pluripotency-associated epigenotype that is necessary to initiate reprogramming. We have demonstrated the functional importance of the 3′ region of *Oplr16* for its interaction with both SMC1 and TET2. The blast search against the mouse reference genome, however, identifies multiple high homology sequences within the 3′ region of *Oplr16*, suggesting that this region may be highly conserved among different lncRNAs. Future studies are needed to address if they share a common mechanism in gene regulation. Additionally, it is still unclear if the intrachromosomal looping is a primary or secondary effect of the *Oplr16*-SMC1 interaction.

The role of the cohesion complex SMC1 in orchestrating intrachromosomal interactions, particularly in the regulation of pluripotent genes, has been elucidated using different approaches. For example, knockdown of SMC1with shRNA abolishes the intrachromosomal looping in *Oct4*, and induces the loss of self-renewal in human H14 ESCs (56). However, we still do not know how *Oplr16* coordinates with SMC1 in the looping process. Multiple stem cell core factors and other chromatin factors are also involved in the loop formation. We cannot rule out the possibility that the downregulation of these factors in the *Oplr16* knockdown cells may also trigger the loss of intrachromosomal looping.

It should also be emphasized that *Oplr16* is not the sole factor that regulates the *Oct4* gene in reprogramming. Overexpression of *Oplr16* in fibroblasts did not translate into the full activation of *Oct4*. Other factors, including stem cell core factors, chromatin factors, and other *Oct4*-interacting lncRNAs, may be also required to coordinate the reprogramming process. Future studies are needed to examine if *Oplr16* is able to corporate with other factors in enhancing reprogramming, and if *Oplr16* can replace one or more factors in the conventional *Oct4-Sox2-Klf4-c-Myc* cocktail. Hopefully, a knockout mouse model will be able to further address the role of *Oplr16* in embryo development.

In a DOX-inducible *Oct4* ES cell model, Niwa *et al.* found that *Oct4* was required to sustain pluripotency and self-renewal. Ectopic expression of *Oct4* alone, however, was not sufficient to maintain undifferentiated phenotypes induced by LIF-withdrawal (57). In our study, however, we found that the *Oct4*-interacting lncRNA *Oplr16* alone was able to delay LIF withdrawal-induced differentiation. Thus, epigenetic regulation of the *Oct4* promoter alone may not completely account for the roles of *Oplr16*. The genome-wide target mapping by RAT-seq shows that *Oplr16* targets multiple signal pathways in development and cell differentiation. Thus, it is possible that *Oplr16* may control pluripotency through multiple mechanisms in addition to the *Oct4* pathway.

In summary, this study identifies *Oplr16* as a novel pluripotency-associated lncRNA in the mouse. Genetic depletion of *Oplr16* causes E14 cells to differentiate and lose pluripotency. *Oplr16* maintains stem cell pluripotency through multiple epigenetic mechanisms, including the activation of endogenous stem cell core factor genes, coordination of intrachromosomal looping, regulation of the *Tet2* gene, and recruitment of TET2 to induce DNA demethylation. Thus, this lncRNA functions as a chromatin modulator in the regulatory network of stem cell pluripotency and reprogramming of somatic cells. Further studies are needed to explore its potential for use in regenerative therapies. In addition to *Oplr16*, the CRIST-seq assay has identified dozens of lncRNAs that interact with the *Oct4* and/or *Sox2* promoters. It would be interesting to learn how these lncRNAs cooperate each other in the maintenance of pluripotency. For example, *Peblr20* (*Pou5F1*enhancer binding lncRNA 20) binds to *Oct4* enhancers, where it recruits TET2 demethylase, leading to the activation of the enhancer RNA (eRNA) pathway (58). Similarly, the genome-wide interaction target mapping by RAT-seq shows that *Oplr16* binds to many targets involved in multiple signal pathways that are related to development and cell differentiation. It is well known that suppression of cell cycle regulator and senescence pathway genes, like p16, p21 and p53, promotes pluripotent reprogramming (45,59,60). Valproic acid, a histone deacetylase inhibitor, enhances reprograming partially through the suppression of the senescence pathway (25,61). In this study, we also observed that *Oplr16* downregulates the senescence pathway in MEF cells. On the other hand, knockdown of *Oplr16* upregulates the expression of p16, p21, and p53. Clearly, *Oplr16* may control pluripotency through multiple mechanisms. Epigenetic regulation of the *Oct4* promoter may just be a portion of the roles of *Oplr16*. Future studies are needed to detail these *Oplr16* non-*Oct4* pathways in stem cells and to discover how they work coordinately in the regulation of DNA methylation, histone modifications, and 3D chromatin structures.

## DATA AVAILABILITY

As previously reported (21,22), the CRIST-seq, RAT-seq and RNA-seq data generated in this study have been deposited in NIH GEO databases under accession numbers GSE107945, GSE101765 and GSE116605, respectively. The datasets are available at https://www.ncbi.nlm.nih.gov/geo/query/acc.cgi?acc=GSE107945; https://www.ncbi.nlm.nih.gov/geo/query/acc.cgi?acc=GSE101765; and https://www.ncbi.nlm.nih.gov/geo/query/acc.cgi?acc=GSE116605.

## Supplementary Material

gkaa097_Supplemental_File
