# Supplementary material for: Oplr16 serves as a novel chromatin factor to control stem cell fate by modulating pluripotency-specific chromosomal looping and TET2-mediated DNA demethylation
Source: Nucleic Acids Res. 2020 Feb 14;48(7):3935–48. doi: 10.1093/nar/gkaa097 (PMC7144914; doi:10.1093/nar/gkaa097)
Supplement: gkaa097_Supplemental_File [file gkaa097_supplemental_file.pdf]

Oplr16 serves as a novel chromatin factor to control stem cell fate by modulating pluripotency-specific chromosomal looping and TET2-mediated DNA demethylation

Lin Jia[1,2*], Yichen Wang[1,2*], Cong Wang[1,2], Zhonghua Du[1], Shilin Zhang[1], Xue Wen[1], Lei Zhou[1], Hui Li[1], Huiling Chen[2,3], Dan Li[1], Songling Zhang[1], Wei Li[1], Wei Xu[1#], Andrew R. Hoffman[2*], Jiuwei Cui[1*], Ji-Fan Hu[1,2*]

[1] Key Laboratory of Organ Regeneration and Transplantation of Ministry of Education, Stem Cell and Cancer Center, First Hospital, Jilin University, Changchun, Jilin 130061, P.R. China

[2] Stanford University Medical School, VA Palo Alto Health Care System, Palo Alto, CA 94304, USA

[3] Department of Endocrinology, Xiangya Hospital, Central South University, Changsha, Hunan, P.R. China

* These authors contributed equally to the work.

SUPPLEMENTAL TABLES

Table S1. Oligonucleotide primers used for PCR

ID	Oligo Name	Oligo sequence	Product size
RT-PCR			
Oplr16	JH4744	TCAAGCATTACGATGTTCCTCA	125bp
	JH4745	GAGCTCCGGGGTACTGGTTAG	
Oct4	JH116	CAATGCCGTGAAGTTGGAGAAG	179bp
	JH117	GGCTGAACACCTTTCCAAAGAGA	
Sox2	JH118	GGTTACCTCTTCCTCCCACTCCAG	193bp
	JH119	TCACATGTGCGACAGGGGCAG	
Nanog	JH120	TCTCCTCCATTCTGAACCTGAGC	150bp
	JH121	TGCTGGGATACTCCACTGGTGCT	
U6	JH4061	GTGCTCGCTTCGGCAGCACATATAC	102bp
	JH4062	ATATGGAACGCTTCACGAATTTGCG	
β-Actin	J880	CAGGTCATCACCATTGGCAATGAGC	135bp
	J881	CGGATGTCCACGTCACACTTCATGA	
Oplr16 shRNA			
shOplr16-1: #1		TGCACTTCATGGTAACTTTATACAG	
#3		CTGGGATCAGCATTTGACAAATGTC	
shOplr16-2: #2		CATACTGAAGATGTATGTCTACATA	
#4		TGGATACAATGGATTTGGTAAGATC	
Control (shCT)		GCAGCAACTGGACACGTGATCTTAA<br>TGAAATGTACTGCGCGTGGAGACTA	
Oplr16 overexpression			
Oplr16	JH4989	TCTCGATATCCTCGAGTGAAAATGT AAACGAGGAAAATACCT	739bp
	JH4990	AATCGAAGAATTCGCCCTGGTGTTC CCCTGTACTGG	
RAT primers			
Forward	JH4989	TGAAAATGTAAACGAGGAAAATAC CT	
Reward A	JH4745	GAGCTCCGGGGTACTGGTTAG	600bp
Reward B	JH4919	CATTTGTCAAATGCTGATCCCAG	462bp

Reward C	JH4918	CTACTGCTGCATGCAAAGGCT	190bp

Oct4 Binding

5' Control	JH5813	AGGGCTGTGCCCAGATGAGC	143bp
	JH5814	ACAAGGCTCTGGCCATCCTG	
5' Enhancer	JH4350	CAGATGAGCCAACAGGTCTG	125bp
	JH4351	CAGCAACTTTGTCTGAAGTCC	
Promoter 1	JH4352	AGTTGTCCCCAGGGGAGCCAT	140bp
	JH4353	AAGGGGCCTGGGAGGGACTG	
Promoter 2	J648	CAGAGGATGGCTGAGTGGGCTGTA	123bp
	J649	CACCCCTGCCTTGGGTCACCG	
Exon 1	JH4664	TCAGGTTGGACTGGGCCTAG	121bp
	JH4665	GCGGTCGGCACAGGGCTCAG	
Intron 1	JH4355	GGACTAGAACCCAGAATTGCA	126bp
	JH4356	GTACAGACAGTGATGGCATG	
3' Control	JH5815	GAGCTGCAGGGACCGTAGAC	149bp
	JH5816	CACTTGCTTGTCCCAACTACG	

Sox2 Binding

5' Control	JH4405	CATAAGTAGTTCCCCACTGA	135bp
	JH4406	AATGCAAGGGCTTTGCACGC	
5' Enhancer	JH4454	GCAGTGAGAGGGGTGGACTA	135bp
	JH4455	CAGGCTTGGCTGTTATTGTC	
Promoter	JH4373	GAGCCAATATTCCGTAGCATG	196bp
	JH4374	CGCTGGGGAACCTTTGTATC	
Exon 1	J652	GAAGTTTGGAGCCCGAGGCTTAAG	164bp
	J653	TGCACGGCCCTGCGCGGAGATCTG	
Exon 2	JH5054	GAAGCGGCCGTTCATCGACG	229bp
	JH5055	GTGCGCGTAGCTGTCCATGC	
3' Enhancer	JH4375	GCAGATGAGATACTGCGCCA	152bp
	JH4376	CAGAACGCTCGGCGCGTCTAC	
3' Control	JH4377	GTCTATTCTCCCAGCTGCTCA	129bp
	JH4378	TCAGGCCCCAGGACCACACT	

DNA methylation

CpG	JH4882	GTGGGATTGGGGAGGGAGAGGTGA A	220bp
	JH4883	TCCAAACCCACCTAAAAACCCTTAA	
Mutant *Oplr16*	JH4989	TCTCGATATCCTCGAGTGAAAATGT AAACGAGGAAAATACCT	500bp
	JH6111	cagAatcgaaGAATTCCATTTGTCAAAT GCTGATCCCAG	

Primers for RNA fragments of Oplr16

Oplr16-A	JH4991	GATCTGCGTAATACGACTCACTATAG GGAGATGAAAATGTAAACGAGGAAA ATAC	600bp
	JH4745	GAGCTCCGGGGTACTGGTTAG	
Oplr16-B	JH4991	GATCTGCGTAATACGACTCACTATAG GGAGATGAAAATGTAAACGAGGAAA ATAC	462bp
	JH4919	CATTTGTCAAATGCTGATCCCAG	
Oplr16-C	JH4991	GATCTGCGTAATACGACTCACTATAG GGAGATGAAAATGTAAACGAGGAAA ATAC	190bp
	JH4918	CTACTGCTGCATGCAAAGGCT	

Primers for Oplr16 mapping

Oplr16-1	JH4989	TGAAAATGTAAACGAGGAAAATAC CT	190bp
	JH4918	CTACTGCTGCATGCAAAGGCT	
Oplr16-2	JH6082	GCACATACTGAAGATGTATGTCTAC	135bp
	JH6083	GCAAACCTTTACCCTCTGACT	
Oplr16-3	JH6085	CCATACTGCTAAGAAACACCATG	124bp
	JH6086	GTGATATACAGTCTTTAACACACTG	
Oplr16-4	JH6084	GTCTTTGAAAACCCGAGAGAACT	122bp
	JH6111	CATTTGTCAAATGCTGATCCCAG	
Oplr16-5	JH4744	TCAAGCATTACGATGTTCCTCA	125bp
	JH4745	GAGCTCCGGGGTACTGGTTAG	
Oplr16 3’-1	JH6090	TGGGATCAGCATTTGACAAATGTC	159bp
	JH4745	GAGCTCCGGGGTACTGGTTAG	
Oplr16 3’-2	JH6090	TGGGATCAGCATTTGACAAATGTC	107bp
	JH6089	GTTCCCTTTAGCTCCTTGGGTA	
Oplr16 3’-3	JH4744	TCAAGCATTACGATGTTCCTCA	125bp
	JH4745	GAGCTCCGGGGTACTGGTTAG	
Oplr16 3’-4	JH4744	TCAAGCATTACGATGTTCCTCA	73bp
	JH6089	GTTCCCTTTAGCTCCTTGGGTA	

Oct4-dCas9 gRNAs

pOct4-1	GAACATTCAATGGATGTTTT
pOct4-2	GTGTGAGGGGATTGGGGCTC
5’-CT	GAAGTGGGATGATCCTCTGA

SUPPLEMENTARY FIGURES

Figure S1. Specific expression of *Oplr16* in iPSCs

A. Differential expression of *Oplr16* between iPSCs and fibroblasts. The IGV signals of *Oplr16* from RNA-seq.

B. Specific expression of *Oplr16* in E14 embryonic pluripotent stem cells. Little or no expression *Oplr16* observed in nine tissues. ** $p<0.01$ as compared with tissues.

Figure S2. *Oplr16* interacted with *Oct4* promoter by chromatin-RNA *in situ* reverse transcription trap sequencing (CRIST-Seq).

A. CRIST-Seq assay. IPSC cells were transfected by dCas9-sgRNA lentiviruses. After puromycin selection, cells carrying the CRISPR Cas9 *Oct4*-gRNA cassette were treated with formaldehyde to fix the Cas9 gRNA-*Oct4* promoter chromatin complex structure. The promoter-interacting RNAs were *in situ* reverse transcribed into biotin-cDNAs in the isolated nuclei with biotin-dCTP. The promoter chromatin-cDNA complex was then isolated by Cas9 immunoprecipitation and was then purified from genomic DNA by biotin-streptavidin bead purification. The CRIST-captured cDNAs were used for library construction. Illumina sequencing was used to identify lncRNAs that interact with the *Oct4* promoter.

B. Quantitative PCR of *Oplr16* enrichment in CRIST-seq library products. IgG Ctl: IgG immunoprecipitation control; Cas9: the catalytically inactive CRISPR Cas9; Cas9 gRNA: Cas9 guiding RNAs that target the *Oct4* promoter; gCT: a random gRNA control. ** $p<0.01$ as compared with IgG and gCT controls.

Figure S3. Validation of the *Oplr16-Oct4* interaction by ChIRP.

A. The ChIRP (Chromatin Isolation by RNA Purification) assay. Cells are cross-linked to fix the lncRNA-chromatin DNA complex. After cell lysis and sonication, the lncRNA was hybridized to biotinylated oligonucleotide probes. The biotinylated probe/lncRNA /chromatin DNA complex is pulled down with streptavidin beads. The lncRNA-interacting chromatin DNA is extracted and the lncRNA-interacting target signal is quantitated by qPCR.

B. Quantitation of the *Oplr16-Oct4* interaction by quantitative PCR. Oligo-CT: The ChIRP control in which a random biotinylated oligonucleotide DNA probe was used for hybridization; CTL: The assay control in which no biotinylated oligonucleotide DNA probes were used. For comparison, the CTL group was set as 1. ** $p<0.01$ as compared with the control groups.

Figure S4. The *Oplr16* gene sequence.

A. Location of the *Oplr16* gene. LncRNA *Oplr16* is located on mouse chromosome 17.

B. The full-length sequence of lncRNA *Oplr16*.

Figure S5. The *Oplr16* homolog in the rat.

A. Location of the *Oplr16* homolog sequence in rat chromosome 6.

B. The homolog sequence of *Oplr16* in rat.

C. The alignment of the mouse *Oplr16* with the rat homolog.

Figure S6. Activation of the apoptotic pathway in *Oplr16*-knockdown cells.

After *Oplr16* knockdown with lentiviruses, E14 cells were collected and RT-qPCR was used to quantitate the expression of the apoptotic pathway genes, including P16 (A), P21 (B) and P53 (C). shOplr16: Cells treated with shOplr16-1 and shOplr16-2 lentiviruses; shCT: cells treated with the random shRNA lentivirus; Vector: cells treated with the empty vector lentivirus. ** $p<0.01$ as compared with the untreated E14 control cells and vector control cells.

Figure S7. *Oplr16* activates the *Oct4* promoter.

A. Luciferase reporter assay. DNA fragment of *Oct4* promoter and exon was amplified by PCR, and cloned into the pGL3 vector. *Oplr16* vector: the vector containing the full length *Oplr16*; lncR-CT: *Oplr16* antisense lncRNA control; Empty vector: no lncRNA insert. The *Oplr16*-expression vector and *Oct4* promoter-luciferase vector were co-transfected in HEK293T cells. The luciferase activity was quantitated 72 hours after transfection.
B. Activation of the Oct4 promoter by *Oplr16*. For comparison, the value in the Vector group was set as 1. ** $p<0.01$ as compared with the vector control and the LncR-CT control.

Figure S8. *Oplr16* promotes reprogramming.

A. Diagram of pluripotent reprogramming. *Oplr16* overexpression vector and control vectors were examined for their effect on reprogramming of OG2 MEF cells.
B. Reprogramming strategy. OG2 MEF cells were transfected with *Oplr16* and control lentiviruses. After puromycin selection, cells were induced by doxycycline (DOX) to initiate reprogramming.
C. The morphologic change of cells during reprogramming. After doxycycline (DOX) induction, cells were imaged. Note the fast growing iPSC colony (red arrow) in the *Oplr16* treatment group.

Figure S9. *Oplr16* downregulates senescence marker genes in reprogramming. MEF cells were collected on days 0, 3, and 10 after initiation of reprogramming by addition of DOX in the medium. Q-PCR was used to quantitate the expression of senescence maker genes *p16* (A), *p21* (B), *p53* (C), *Apo-J* (D), *Cav1* (E), and *OX-1* (F). * $p<0.05$, ** $p<0.01$ as compared with the vector control and the LncR-CT control.

Figure S10. *Oplr16* rescues the LIF withdrawal-induced defects in E14 cells.

A. The diagram of the rescue assay. E14 cells were transfected with Oplr16 lentiviruses and were selected by puromycin. After selection, E14 cells were collected and seeded at very low density in the medium that lacks LIF. At the end of the assay, stem cell spheroids were imaged, and cells were collected for quantitative PCR of stem cell core factor genes.

B. Stem cell spheroid formation. After withdrawal of LIF in the stem cell culture medium, E14 cells lost pluripotency and became differentiated. Overexpression of *Oplr16*, however, partially rescued the defects caused by LIF-withdrawal, as compared with the vector and random shRNA controls.

C. Expression of stem cell core factor genes. The LIF withdrawal cells were collected for quantitation of stem cell core factors, including *Oct4*, *Sox2* and *Nanog*. Oplr16 rescued the LIF withdrawal-induced downregulation of stem cell core factor genes. ** $p < 0.01$ as compared with iPS and shCT.

Figure S11. *Oplr16* does not activate the *Sox2* promoter.

A. The construct of pSox2-Luciferase plasmid. DNA fragment of *Sox2* promoter and exon was amplified by PCR, and cloned into pGL3 vector.
B. Luciferase assay of the *Oplr16*-*Oct4* promoter co-transfection.
C. The *Oplr16*-*Sox2* interaction by RAT-Seq. The *Oplr16* RAT-seq showed only a tiny peak at the *Sox2* gene locus.
D. Quantitation of the *Oplr16*-*Sox2* interaction by qPCR. Only a small interaction was detected in the *Sox2* exon region. No binding signals of *Oplr16* were detected in other *Sox2* loci.

Figure S12. KEGG pathways of *Oplr16* by RAT-seq.

A. Top 30 pathway enrichment in RAT-Seq.
B. Genetic features of RAT sequencing.
C. Integration of *Oplr16* RAT-Seq data with RNA-Seq data.

Figure S13. GO analysis of *Oplr16* RAT-Seq data.

A. Top 30 GO pathways in RAT-Seq.

B. Consensus motifs of *Oplr16* binding.

Figure S14. The genome-wide target profiling of *Oplr16* by RAT-seq.

The RNA reverse transcription-associated trap Illumina sequencing (RAT-seq) assay was used to map the *Oplr16* interaction target genes. The *Oplr16* interaction network was drawn based on the enrichment fold the top RAT-Seq pathway target genes.

Figure S15. The effect of *Oplr16* on the expression of *Tet1, Tet3* and *Dnmt1*.

A. Tet1 expression. Fibroblasts (FBC) were transfected by lentiviruses. After puromycin selection, cells were collected for qPCR. No significant difference was detected for *Tet1* expression treatment groups.

B. Expression of *Tet3* in *Oplr16* transfected fibroblasts.

C. Expression of *Dnmt1* in *Oplr16* transfected fibroblasts.

Figure S16. Detail mapping of SMC1-binding in the 3'-*Oplr16* fragment.

A. Mapping of the *Oplr16*-TET2 interaction in the 3'-fragment (Oplr16-5). Interaction of the *Oplr16* 3'-frgement with TET2 was further mapped by RNA-chromatin immunoprecipitation (RIP). The TET2-lncRNA chromatin

complex was immunoprecipitated with an antibody against TET2. Immunoprecipitated RNAs were reverse transcribed and the TET2-interacting *Oplr16* was measured by PCR using primers in the *Oplr16* 3'-fragment.

B. Quantitative PCR of the *Oplr16* 3'fragemnt-TET2 interaction. For comparison, the value of the IgG control was set as 1. * $p<0.05$, ** $p<0.01$ as compared with IgG and gCT controls.

Figure S17. The *Oplr16* mutant loses DNA demethylation potential in fibroblasts.

A. Lentiviral vectors of the full length *Oplr16* and its mutant. The *Oplr16* mutant lacks the functional 3'-fragment.

B. Deletion of the 3'-fragment abolishes the activity of *Oplr16* to induce DNA methylation in the *Oct4* promoter. Fibroblasts (FBC) were transfected with *Oplr16* and control lentiviruses and were collected for measurement of DNA methylation by sodium bisulfite sequencing. Solid dot: methylated CpG islands; open dot: unmethylated CpG islands. Numbers under each CpG site: the percentage of methylated CpGs over the CpGs in the sequencing.

Figure S18. Comparison of the *Oplr16* binding and epigenetic modifications at the *Oct4* promoter.

SMC1 ChIA-PET data were downloaded from GEO under accession number GSE57913 (www.ncbi.nlm.nih.gov/geo/). SMC1 ChIA-PET data were analyzed and conversed to BigWig form (1). TET2 data was downloaded as BigWig form from GEO under accession number GSE115972 (www.ncbi.nlm.nih.gov/geo/). ChIP-Seq data for histone modification markers were retrieved from the ENCODE data,

including H3K4me1, H3K4me3, H3K9ac, H3K27ac, and H3K36me3. The IGV (version 2.4.8) was used to compare the overlapping of the data.

Reference

1. Li, G., Fullwood, M.J., Xu, H., Mulawadi, F.H., Velkov, S., Vega, V., Ariyaratne, P.N., Mohamed, Y.B., Ooi, H.S., Tennakoon, C. *et al.* (2010) ChIA-PET tool for comprehensive chromatin interaction analysis with paired-end tag sequencing. *Genome Biol*, **11**, R22.

A. Differential expression of *Oplr16* (RNA-seq)

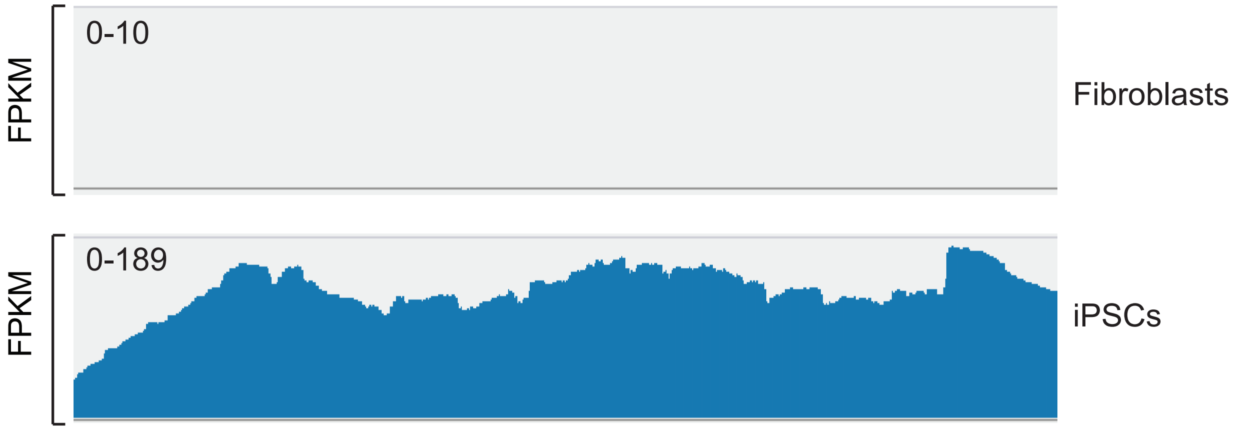

B. Specific expression of *Oplr16* in embryonic stem cells

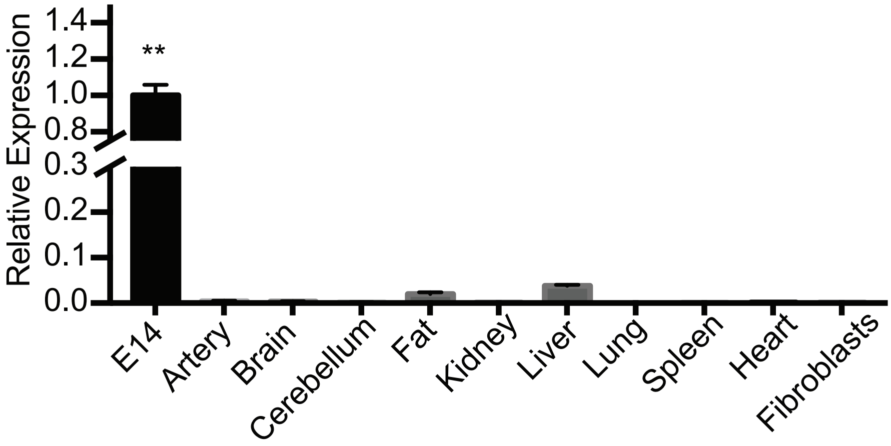

Figure S1. Specific expression of *Oplr16* in pluripotent stem cells

A. CRIST-seq assay

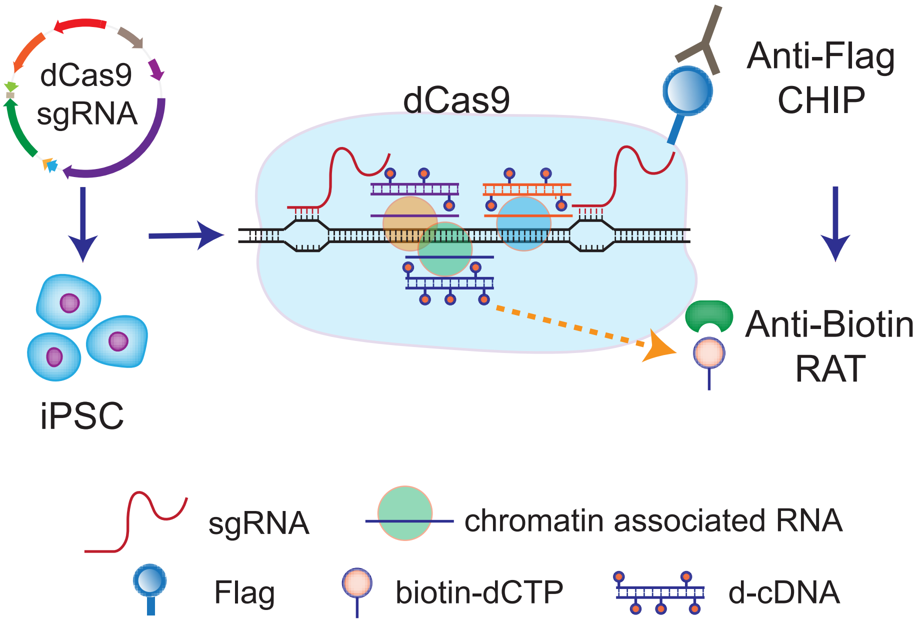

B. Specifity of the *Oplr16* CRIST-seq

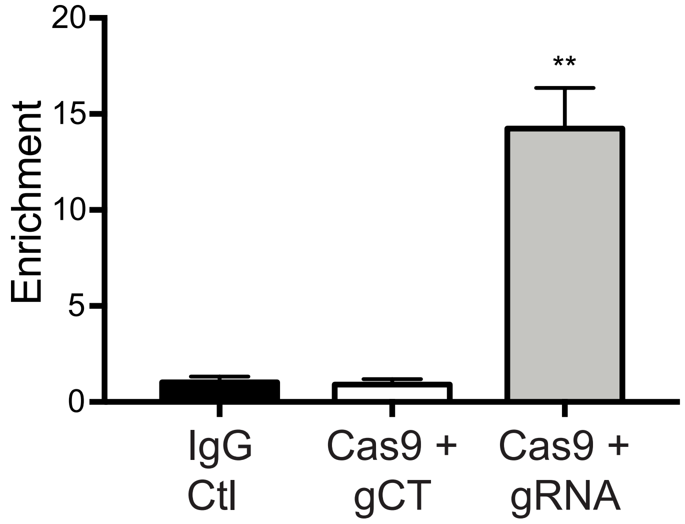

Figure S2. CRIST-seq assay

A. Detection of lncRNA-DNA interaction by ChIRP

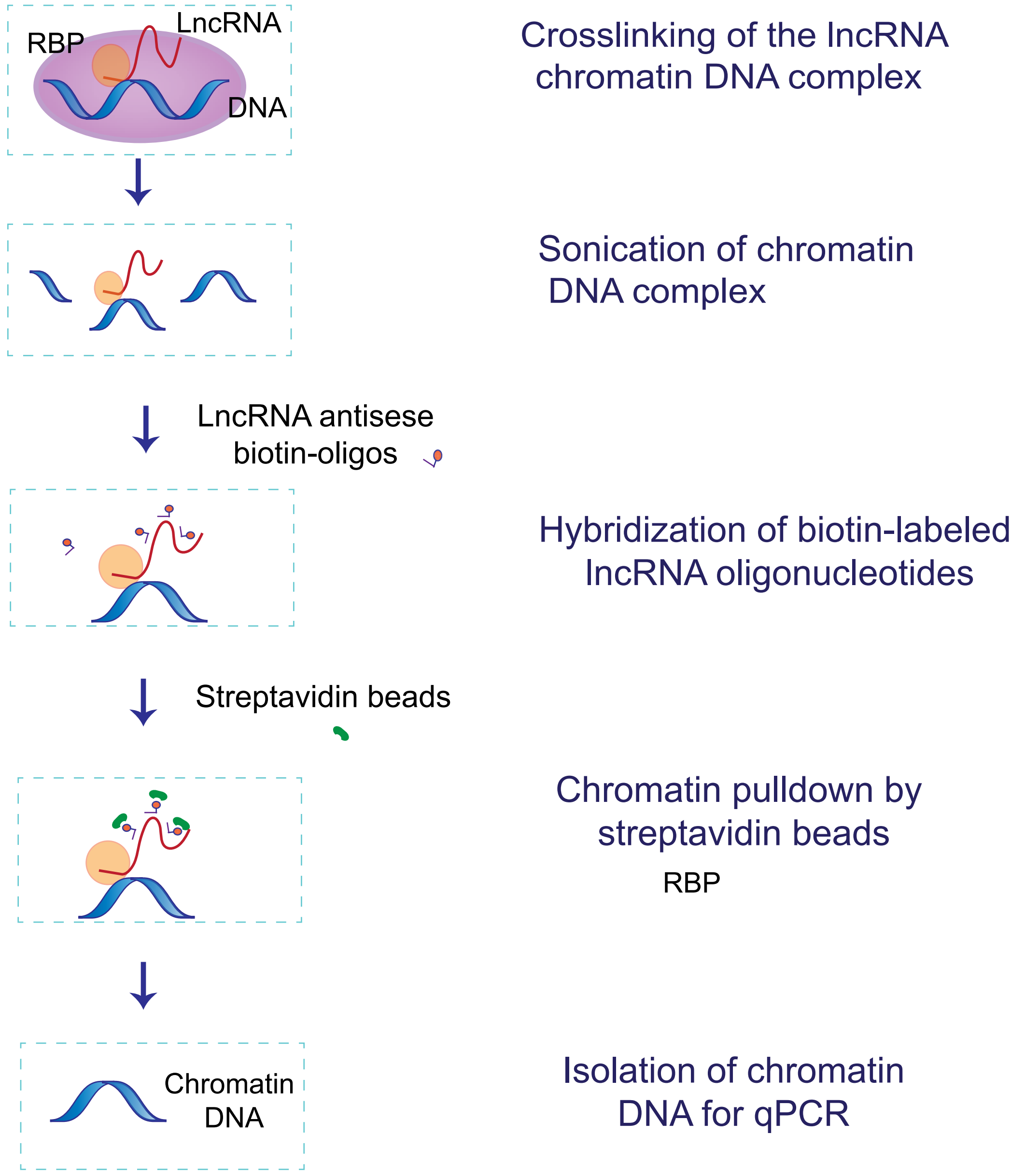

B.*Oplr16-Oct4* interaction

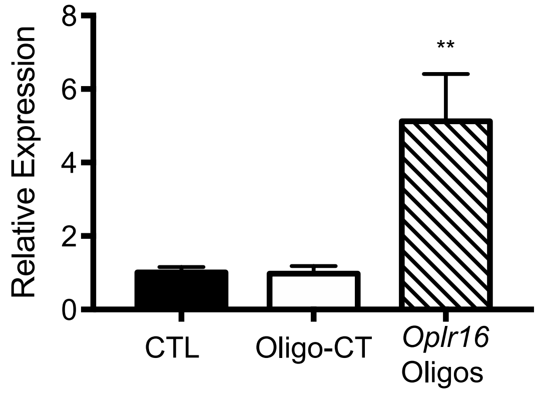

Figure S3. The *Oplr16-Oct4* interaction by Chromatin Isolation by RNA Purification (ChIRP)

A. The *Oplr16* locus

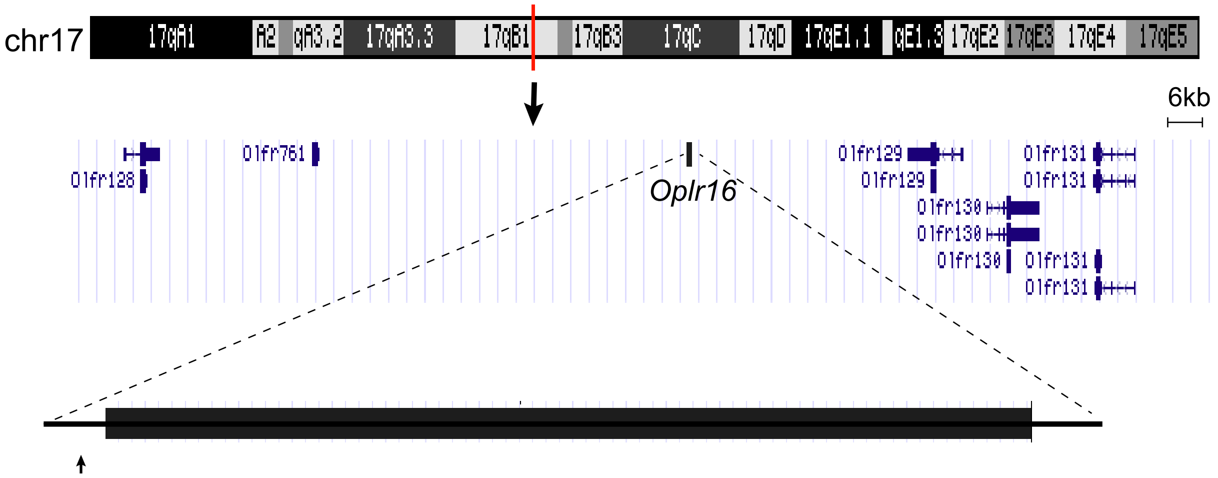

Oplr16 (NONMMUT101010)
chr17:38,014,845-38,015,473

B. *Oplr16* gene sequence

```
TGAAAATGTAAACGAGGAAAATACCTAATTAAAAAAAAAAGAATACATATTGTTTACAAACCCTACAAATG
TAGTCAATGTGATAAAGCCTTTTCACAACACACATATCTATGAATACATAAAAAAGCACATACTGAAGATG
TATGTCTACATACAAGCAATGTGGAAAAGCCTTTGCATGCAGCAGTAGTCATTGAAATTATGAGAAAAAGC
CATACTGCTAAGAAACACCATGAATGTAGTCAGAGGGTAAAGGTTTGCACTTCATGGTAACTTTATACAGG
AGTGAAAAAAAAACTTTATTGTATAATCAGTGTGTTAAAGACTGTATATCACAATAGTCTTTGAAAACCCG
AGAGAACTTATACTAAATGGAATGGATACAATGGATTTGGTAAGATCATTAGTCAACAGTTATTTTCACTT
ACACAAAATTATTCTGGGATCAGCATTTGACAAATGTCTACAATCTATTCAAGCATTACGATGTTCCTCAT
TTTGTTTCTACTTGCAAGCTAGAGAAATTACCCAAGGAGCTAAAGGGAACTGCAACCCTATAGGTAGAACA
ACAATATGAACTAACCAGTACCCCGGAGCTCTTGACTCTAGCTGCATATGTATCAAAAGAT
```

Figure S4. *Oplr16* gene sequence

A. The *Oplr16* homolog in rat

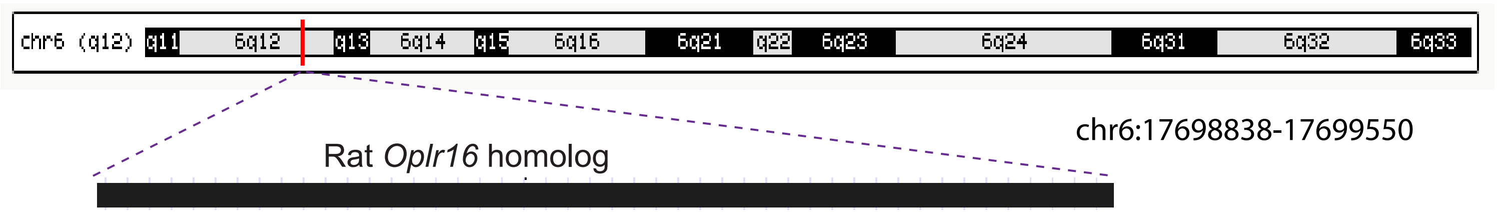

B. Rat *Oplr16* homolog sequence

```
ACCACACCCTTTCCCTCCAAAAGTATAACCTGGAGAGAAACCCTAAGAACGTAACAAGTATG
TTAAAGACTTTGCATTTCTTAGTGGTCTTTAAATACATAAAAGAATAAATACTGGTTACAAA
CCCTACAAATGTAATCAATGTGATAAAGTCTTTTCACCACATACATATCTATGAATACCTGA
AGAACGCATACGGAAGATGTGTGCCTACAGACAAGCAATCTGGGAAAGCCTTTACATGTAGA
AGTAGTCTTTAAAATTATGAGAAAAAGTCATACTGCAGAGAAACACCATGAAGTAGTCAGTG
TAGTAAATGTTTGCACTTCATGGTAACTTTATACAAGAGTAAAACCTATAGTATGTAATCAG
TCTGTTAAAGACTATGTTTATCACAATACTCTTTAAAGATTAAAAGCACTTATACTAAATGG
AGTCTCTAAATATAAAGGATTTGGTAAGATCCTTATTTAACAAACTTATCTTTACTTACACA
AGATTATTGGATAAATATCTGACAAGTGTCTACAATATATTCAAGTATTATGATGTTCCTCC
TTGGTTTTTTTTTTTCTATTTGCAAGCTAATATGGACAAAAGACACCCTTTTATAATTCAAA
ATTCTCTTCAATTACATGAAAAATTCATCTAGGTGTAAAGCTTTTTGGGCATGTATTAGTGC
CTTAGTTTTTGCTGTAAAATAAAACATCTAA
```

C. Alignment of the mouse *Oplr16* and the rat homolog

```
   1' TGA-AAA--- TGTAAACGAG GAAAATACCT AATTAAAAAA AAAAGAATAC ATATTGTTTA  Mouse
      **  **     * *  *       *     **     * * *  *********  *** ** ***
  61" TGTTAAAGAC TTTGCATTTC TTAGTGGTCT TTAAATACAT AAAAGAATAA ATACTGGTTA  Rat

  57' CAAACCCTAC AAATGTAGTC AATGTGATAA AGCCTTTTCA CAACACACAT ATCTATGAAT  Mouse
      ********** ******* ** ********** ** ******* * *** **** **********
 121" CAAACCCTAC AAATGTAATC AATGTGATAA AGTCTTTTCA CCACATACAT ATCTATGAAT  Rat

 117' ACATAAAAAA GCACATACTG AAGATGTATG TCTACATACA AGCAATGTGG AAAAGCCTTT  Mouse
      ** * ** **  * ***** * ******* **  ***** *** ****** ***  *********
 181" ACCTGAAGAA -CGCATACGG AAGATGTGTG CCTACAGACA AGCAATCTGG GAAAGCCTTT  Rat

 177' GCATGCAGCA GTAGTCATTG AAATTATGAG AAAAAGCCAT ACTGCTAAGA AACACCATGA  Mouse
       **** ** * ****** **  ********** ****** *** *****  *** **********
 240" ACATGTAGAA GTAGTCTTTA AAATTATGAG AAAAAGTCAT ACTGCAGAGA AACACCATGA  Rat

 237' ATGTAGTCAG AG-GGTAAAG GTTTGCACTT CATGGTAACT TTATACAGGA GTGAAAAAAA  Mouse
      * ********  *  *****  ********** ********** ******* ** **     ***
 300" A-GTAGTCAG TGTAGTAAAT GTTTGCACTT CATGGTAACT TTATACAAGA GT-----AAA  Rat

 296' AACTTTATTG TATAATCAGT GTGTTAAAGA CT--GTATAT CACAATAGTC TTTGAAAACC  Mouse
      * ** ** *  * ********  ********* **  ** *** ******* ** *** ***
 354" ACCTATAGTA TGTAATCAGT CTGTTAAAGA CTATGTTTAT CACAATACTC TTT-AAAGAT  Rat

 354' CGAGAGAACT TATACTAAAT GGA----ATG GATACAATGG ATTTGGTAAG ATCATTAGTC  Mouse
        * ** *** ********** ***     *   *** ** ** ********** *** *** *
 413" TAAAAGCACT TATACTAAAT GGAGTCTCTA AATATAAAGG ATTTGGTAAG ATCCTTATTT  Rat

 410' AAC--AGTTA TTTTCACTTA CACAAAATTA TTCTGGGATC AGCATTTGAC AAATGTCTAC  Mouse
      ***  * *** * ** ***** ***** **** **   ****  *  ** **** ** *******
 473" AACAAACTTA TCTTTACTTA CACAAGATTA TT---GGATA AATATCTGAC AAGTGTCTAC  Rat

 468' AATCTATTCA AGCATTACGA TGTTCCTCAT T-------TT GTTTCTACTT GCAAGC----  Mouse
      *** ****** ** **** ** ******** * *       **  ****** ** ******
 530" AATATATTCA AGTATTATGA TGTTCCTCCT TGGTTTTTTT TTTTCTATTT GCAAGCTAAT  Rat

 517' -TAGAGAAAT TACCCAAGGA GCTAAAGGGA ACTGCAACCC TATAGGTAGA ACAACAATAT  Mouse
       * ** ***   ** *        ***    * * * *    *  **      ** * **
 590" ATGGACAAAA GACACCCTTT TATAATTCAA AATTCTCTTC AATTACATGA AAAATTCATC  Rat

 576' GAACTAACCA GTACCCCGGA GCTCTTGACT CTAGCTGCAT ATGTATCAAA AGAT        Mouse
       *  *    * *      **    * *          *   *  *   *  ** *
 650" TAGGTGTAAA GCTTTTTGGG CATGTATTAG TGCCTTAGTT TTTGCTGTAA AATA        Rat
```

Figure S5. The rat *Oplr16* homolog

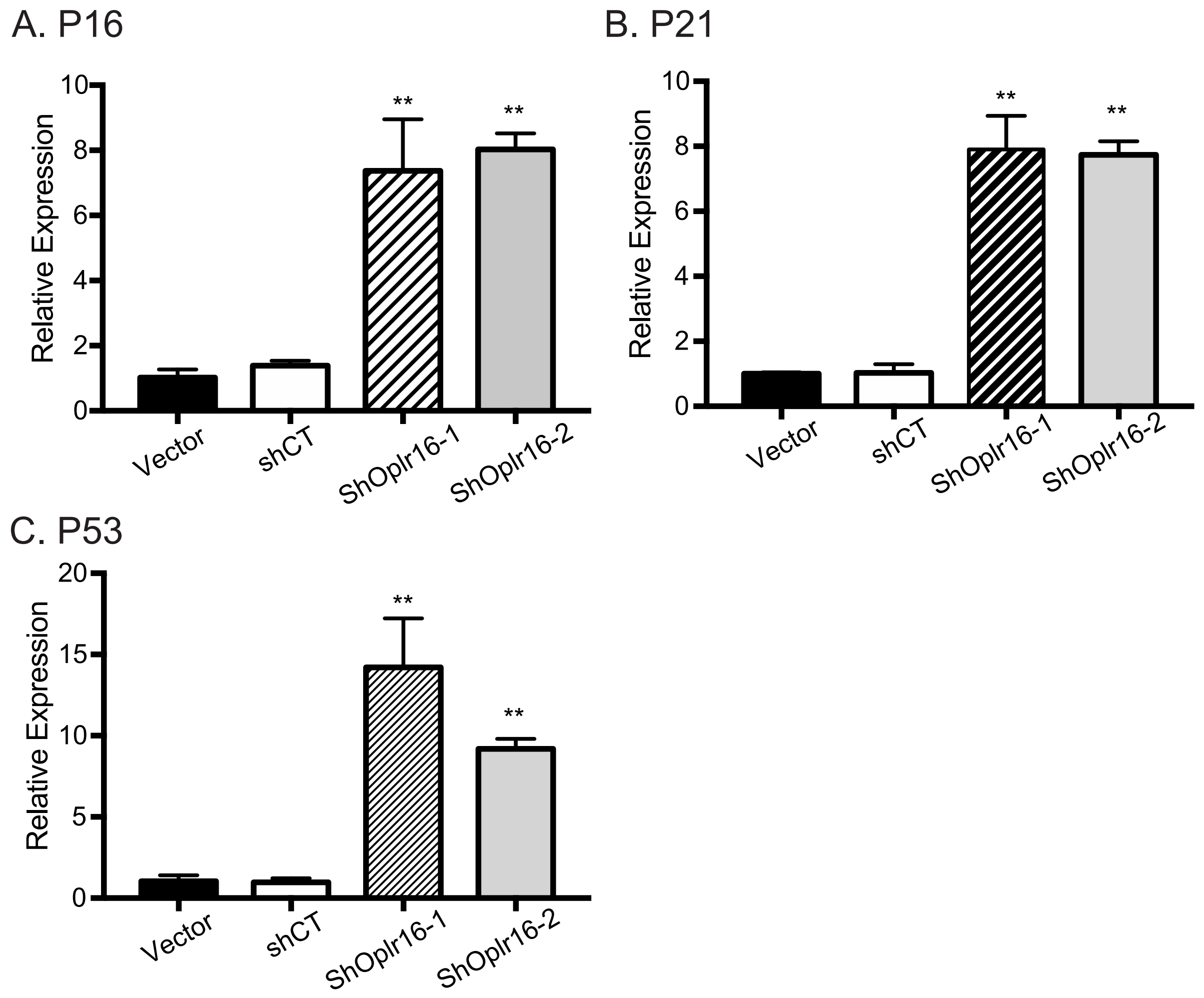

Figure S6. *Oplr16* knockdown activates the senescence pathway

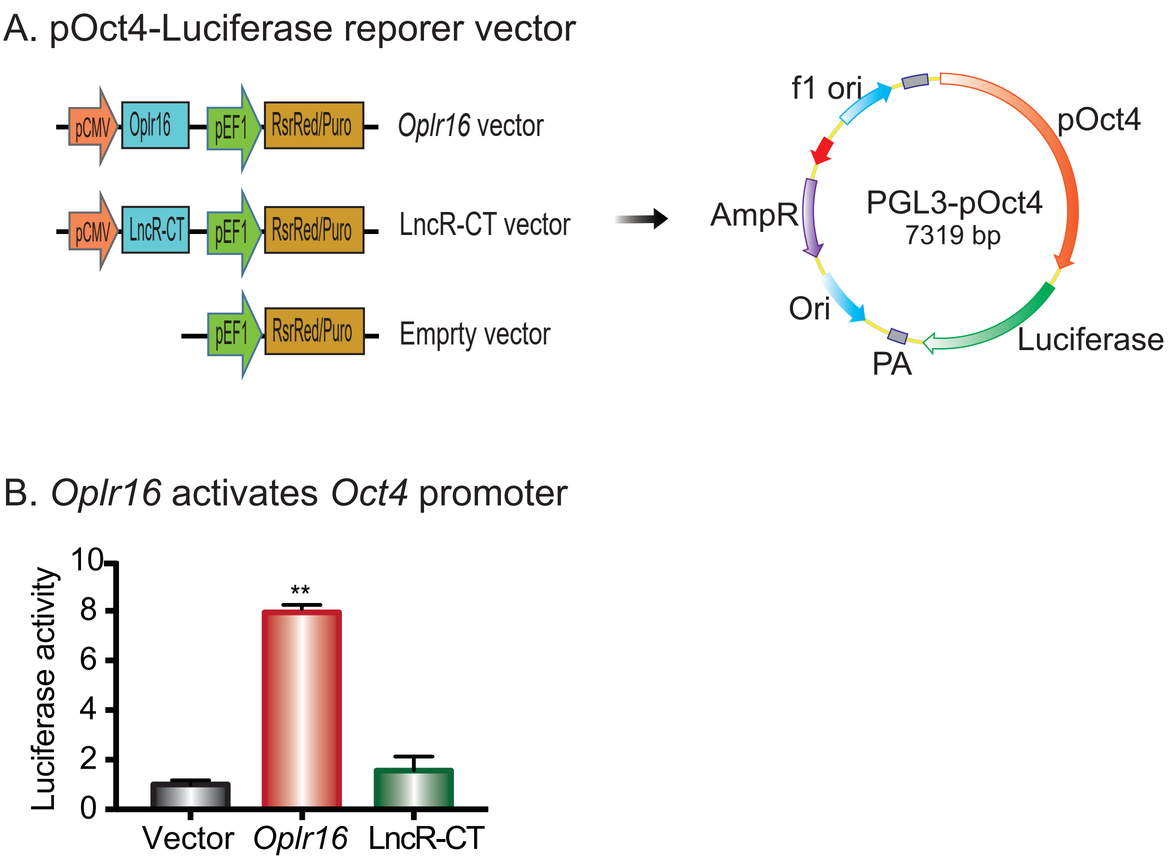

Figure S7. *Oplr16* enhances the *Oct4*-luciferase activity

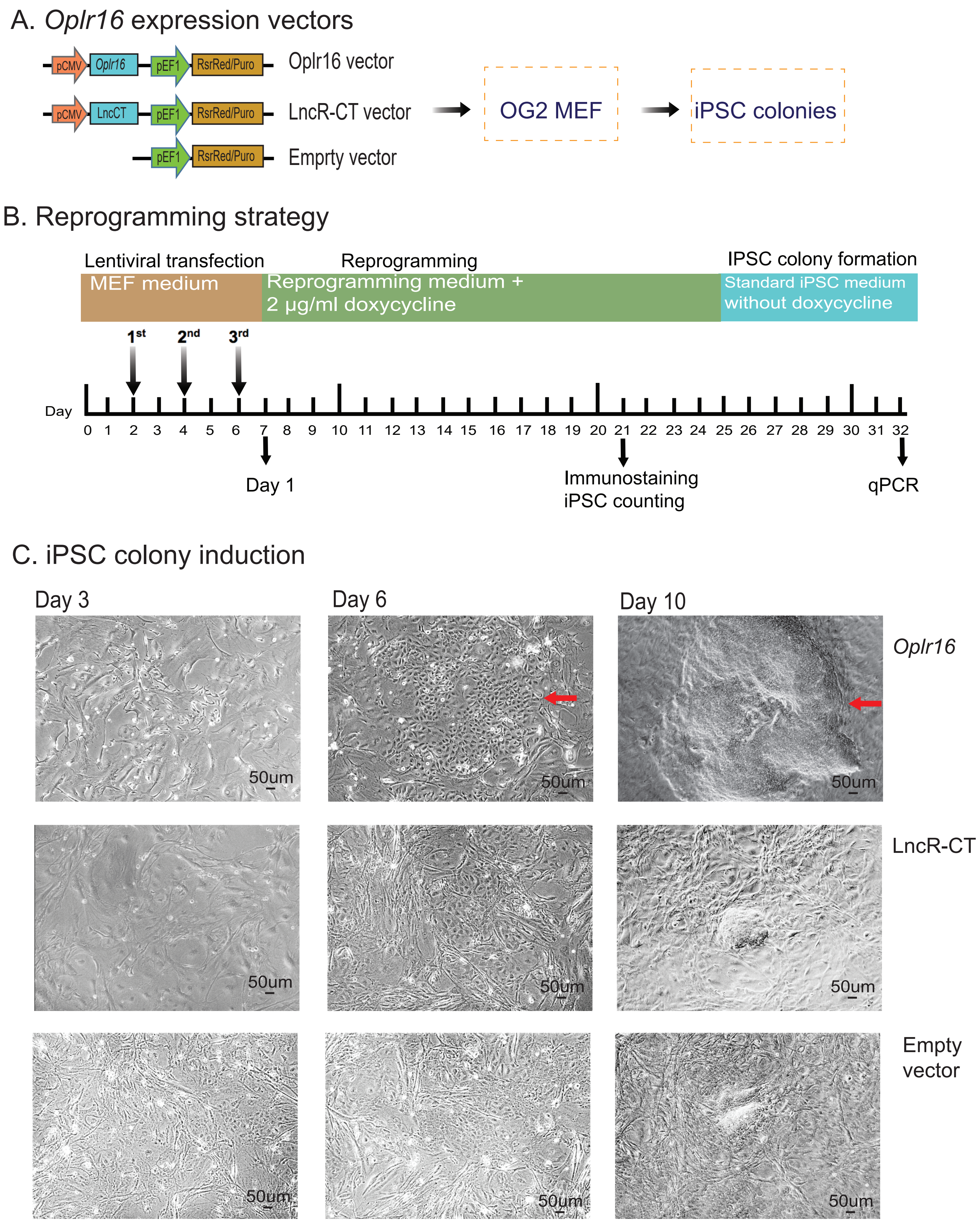

Figure S8. *Oplr16* enhances iPSC colony formation

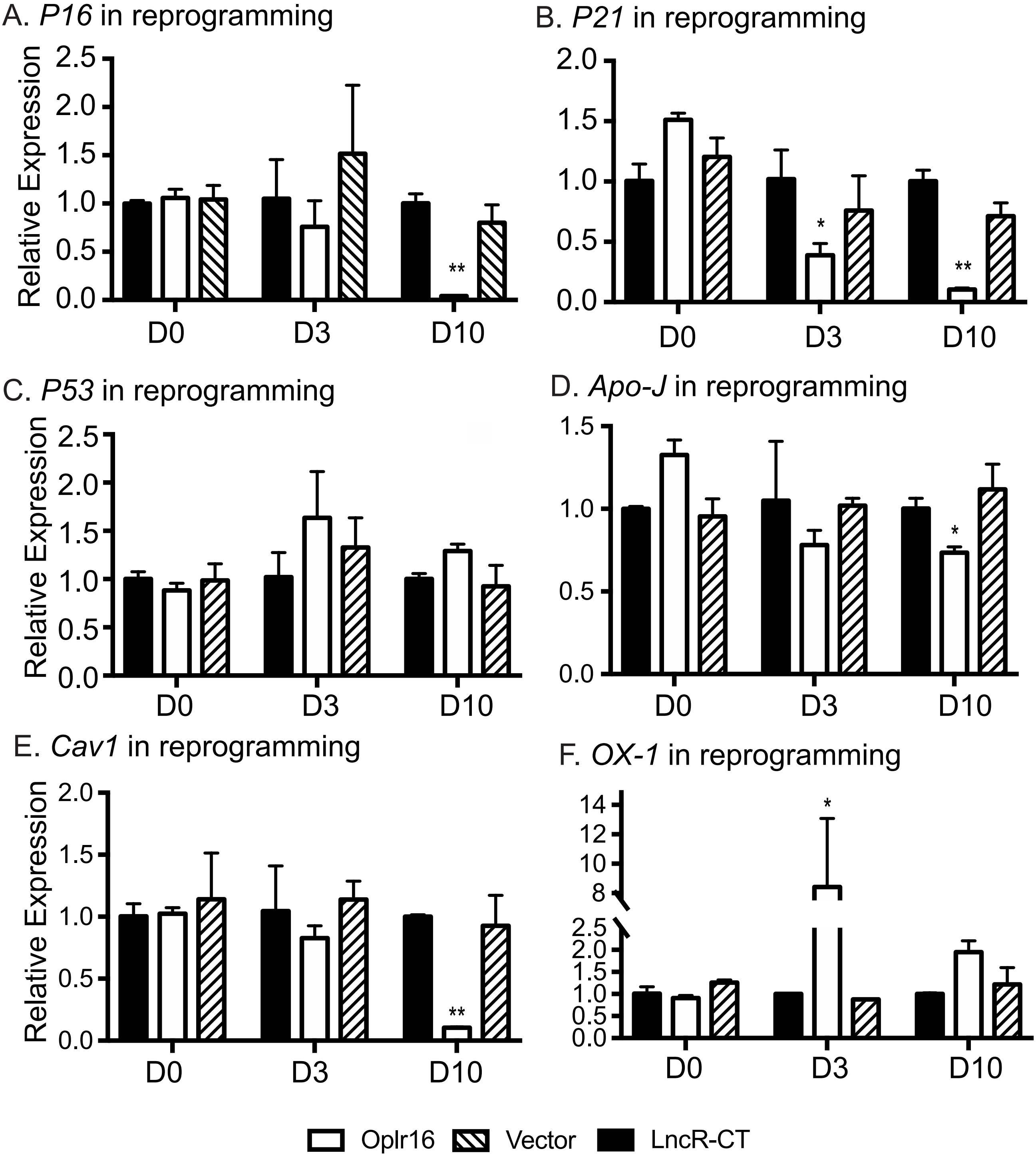

Figure S9. *Oplr16* downregulates senescence markers in reprogramming

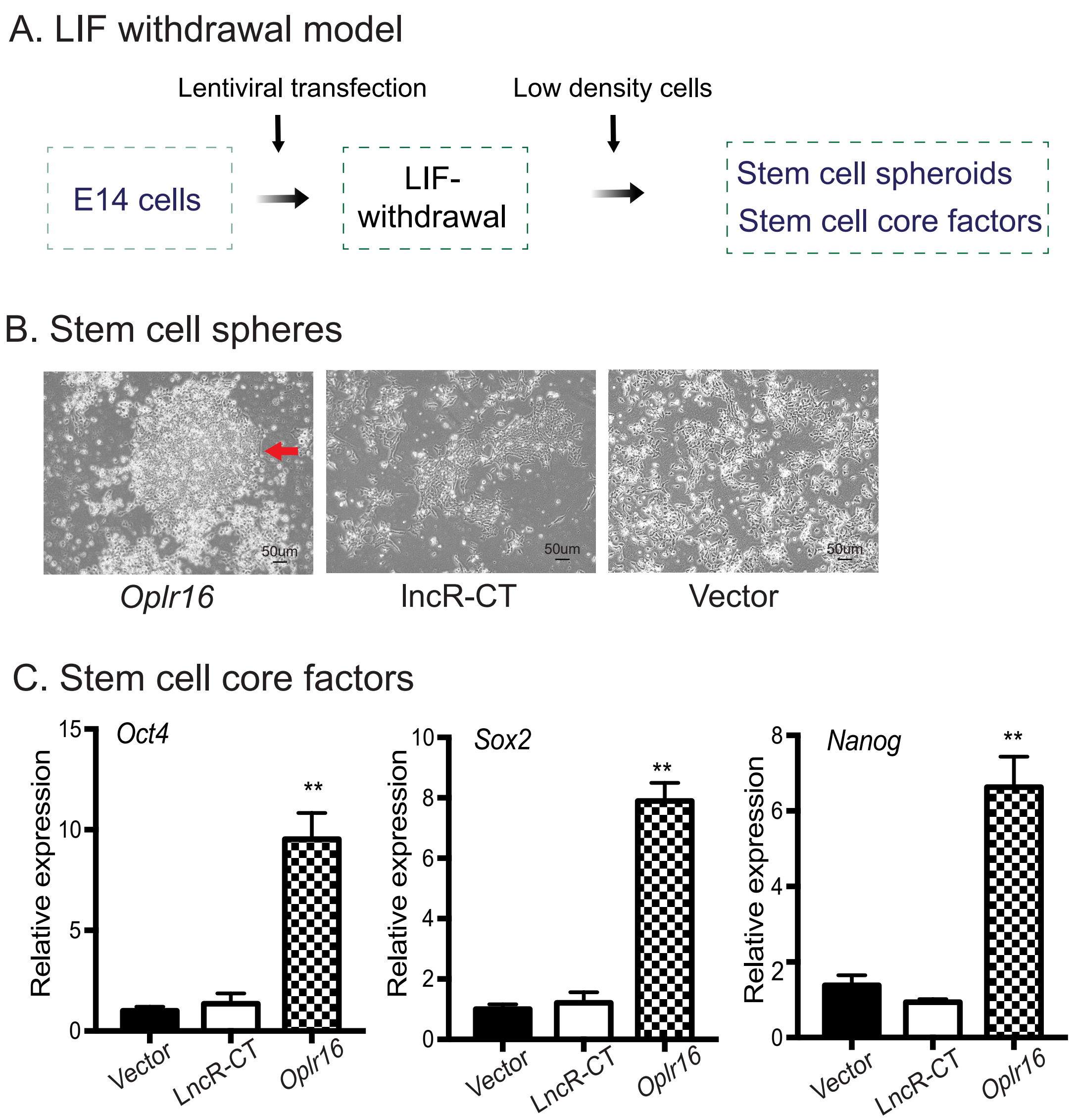

Figure S10. *Oplr16* inhibits LIF withdrawal-induced differentiation

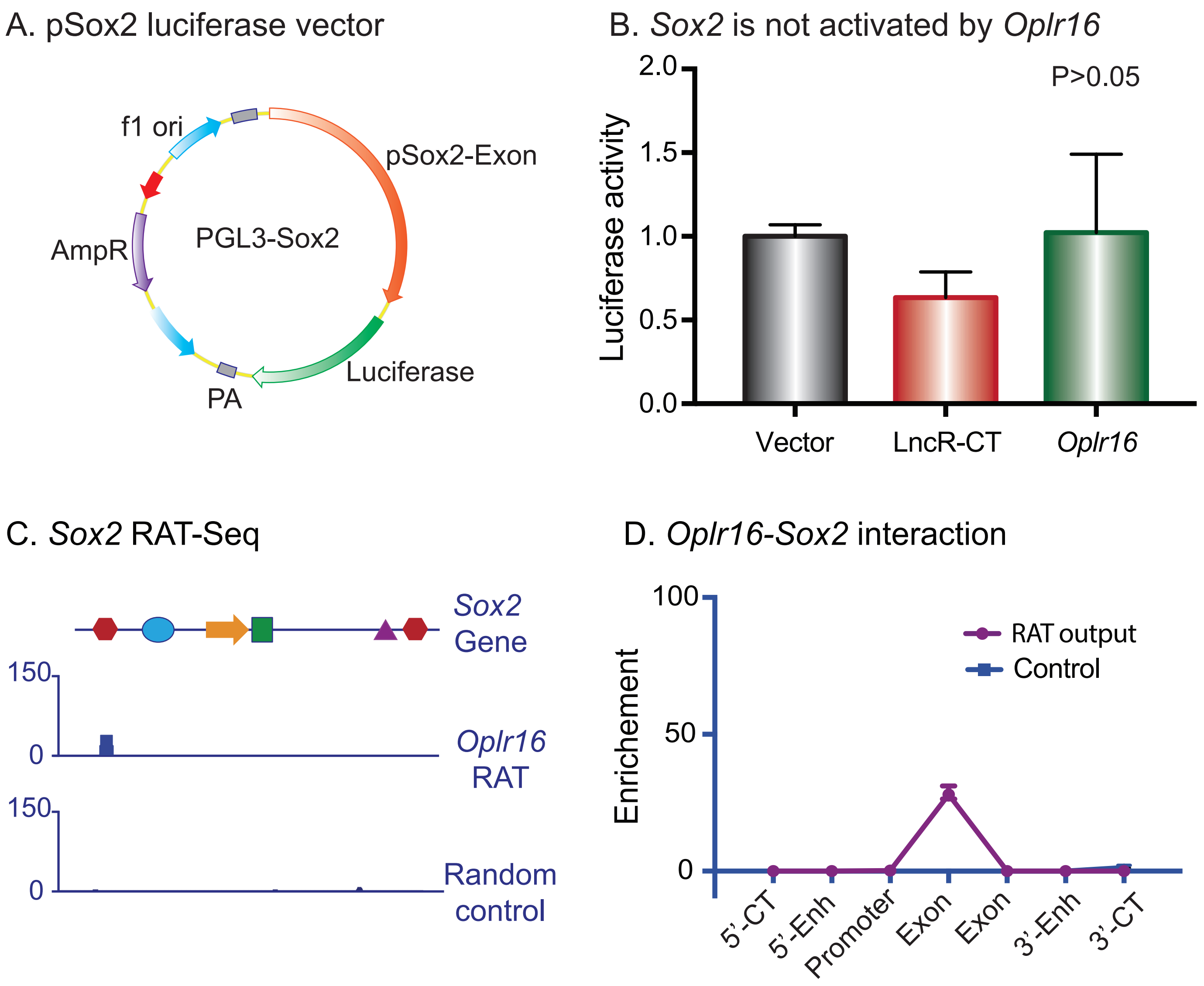

Figure S11. *Oplr16* does not activate the *Sox2* promoter

A. Top 30 KEGG pathway enrichment in RAT-Seq

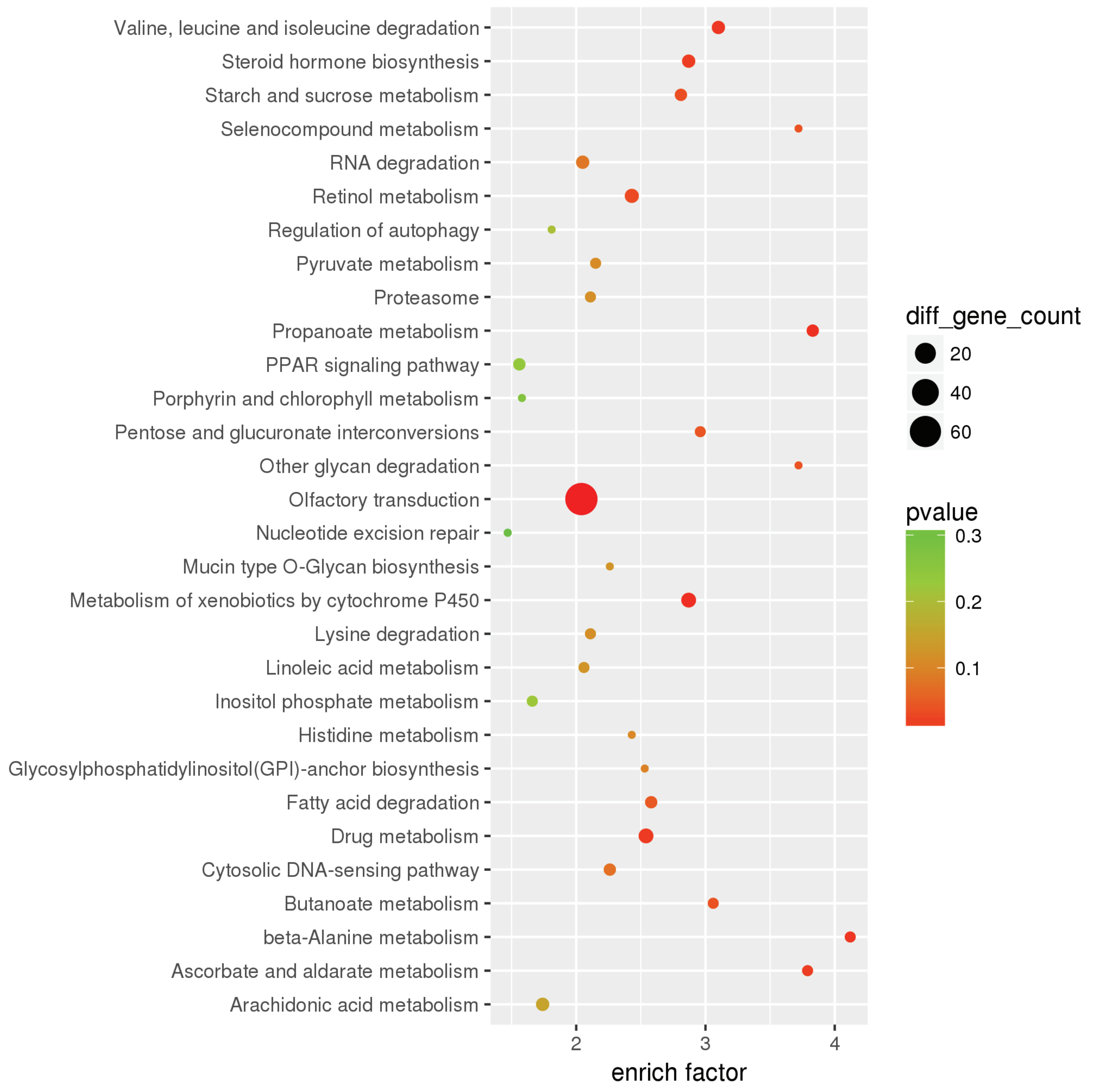

B. Genetic features of RAT sequencing

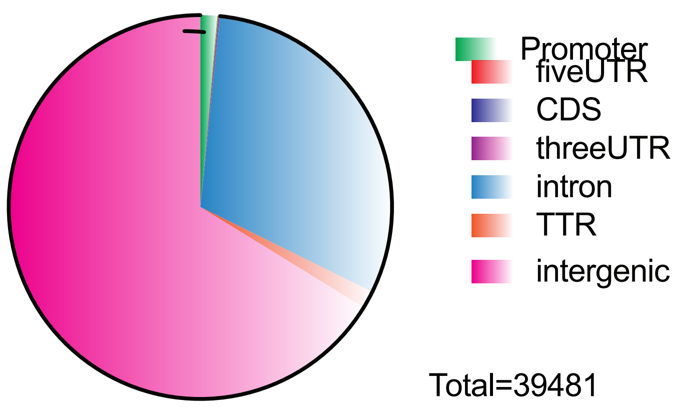

C. Merged genes with iPSC RNA-Seq

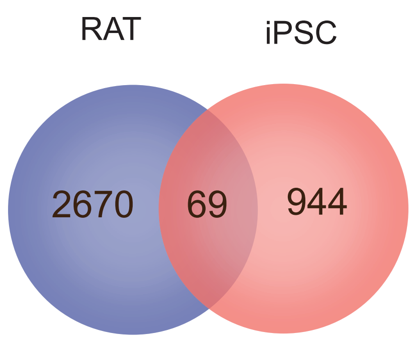

Figure S12. RAT-Seq data of *Oplr16*

A. Top 30 GO pathway enrichment in RAT-Seq

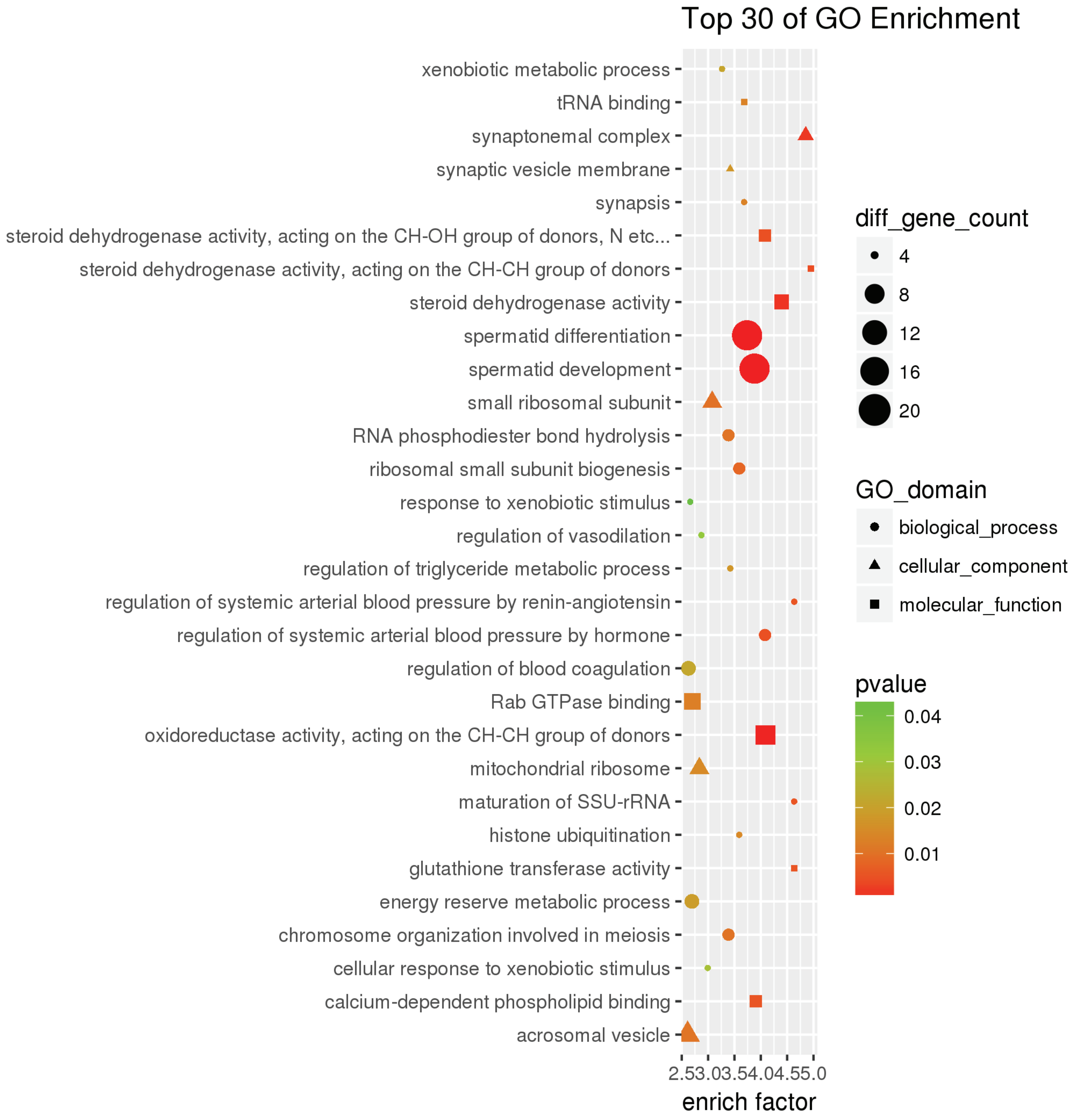

B. Consensus motifs of *Oplr16*

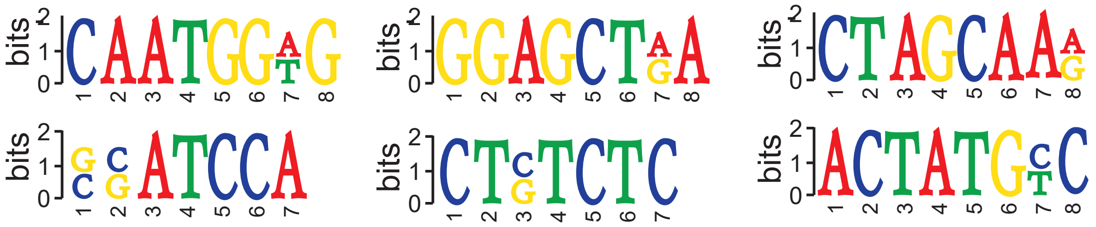

Figure S13. *Oplr16* RAT-seq GO pathways

A. *Oplr16* targets

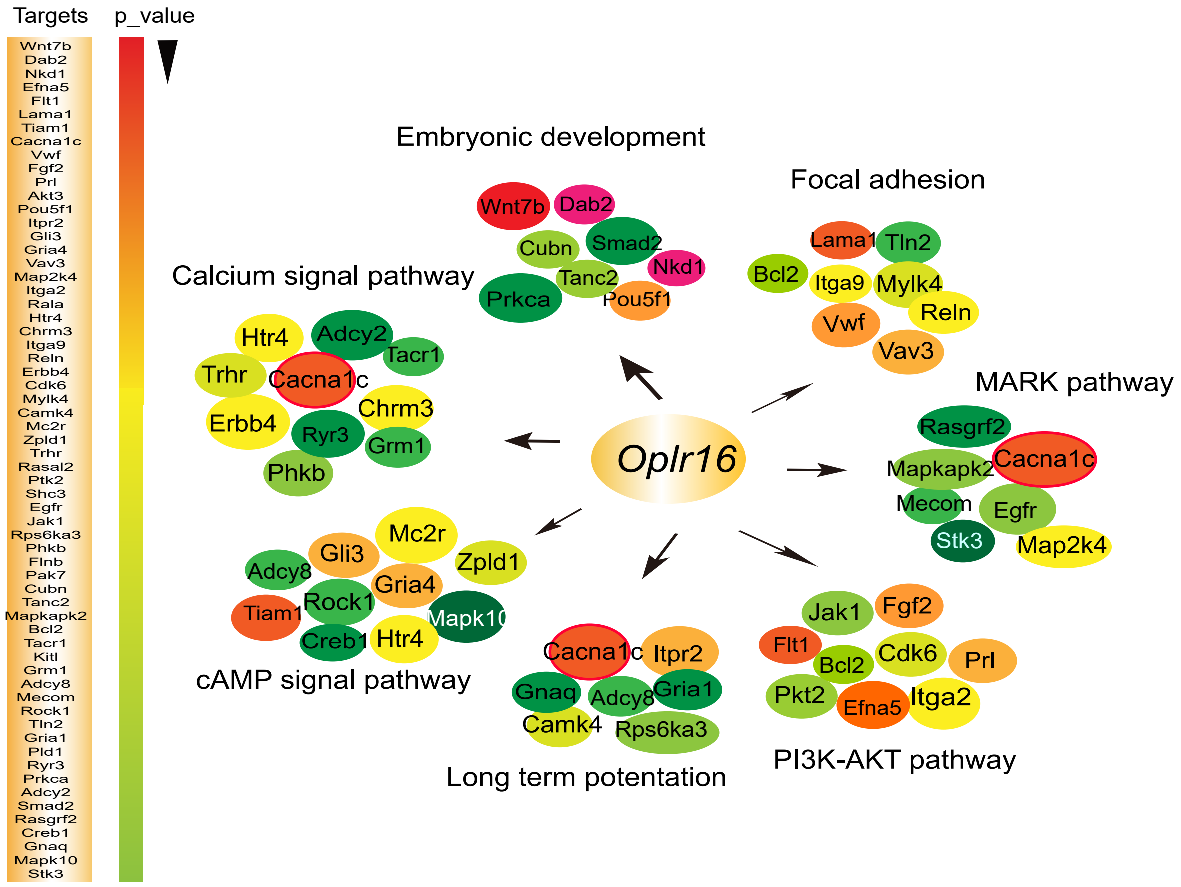

Figure S14. *Oplr16* genome-wide targets by RAT-seq

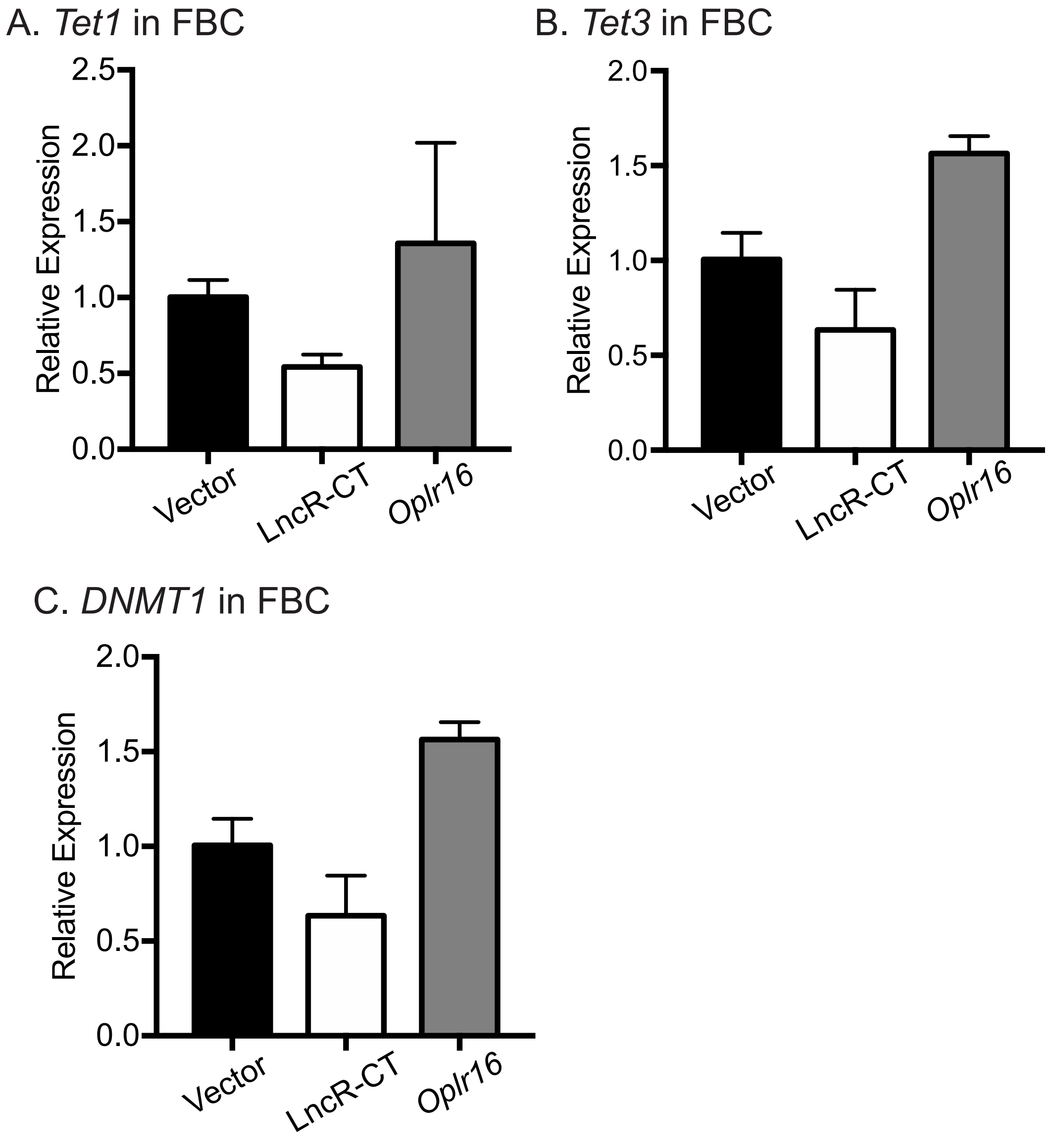

Figure S15. Expression of DNA methylation-related genes in fiblroblasts

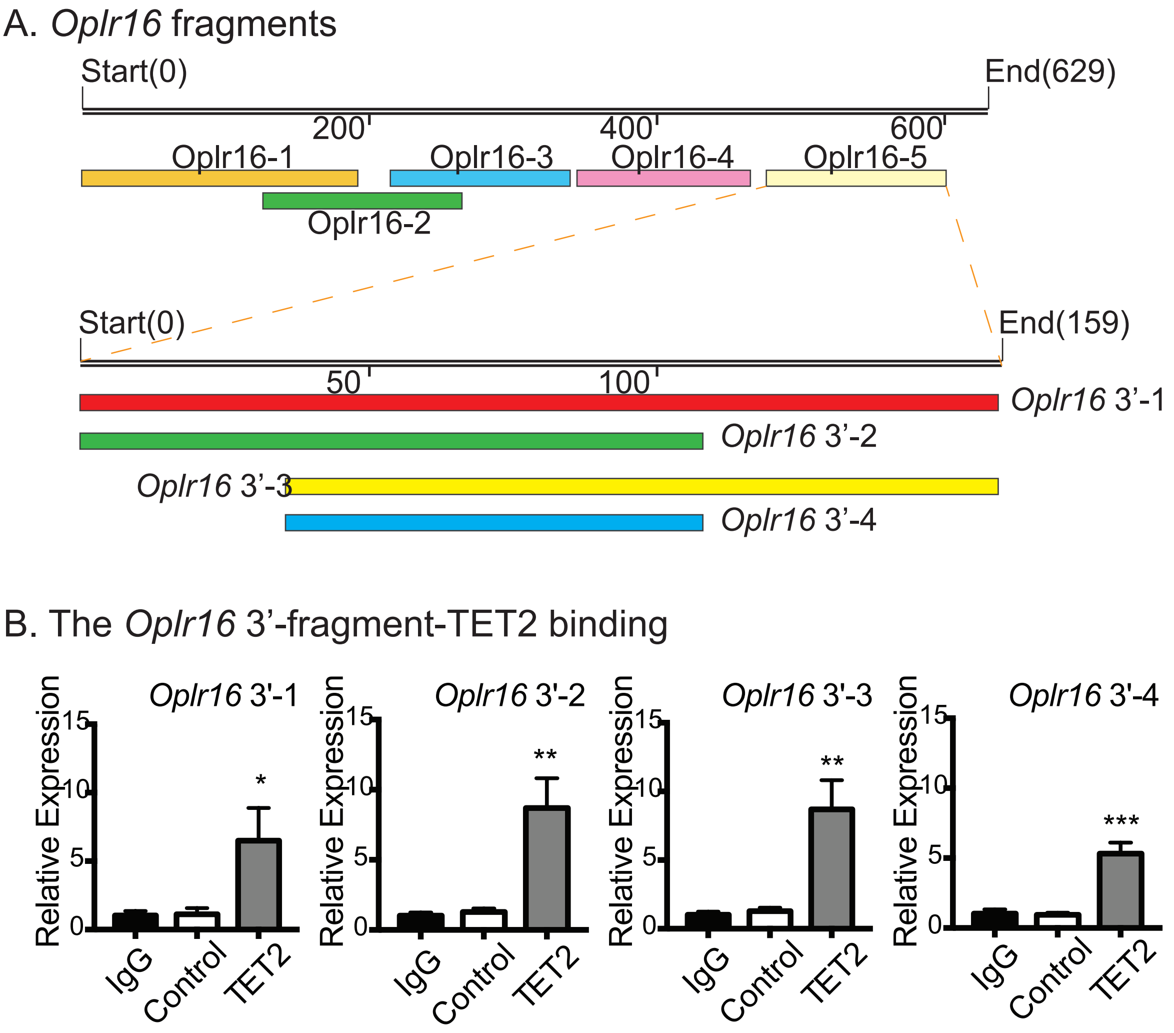

Figure S16. The *Oplr16* 3'-fragment interaction with TET2 DNA demethylase

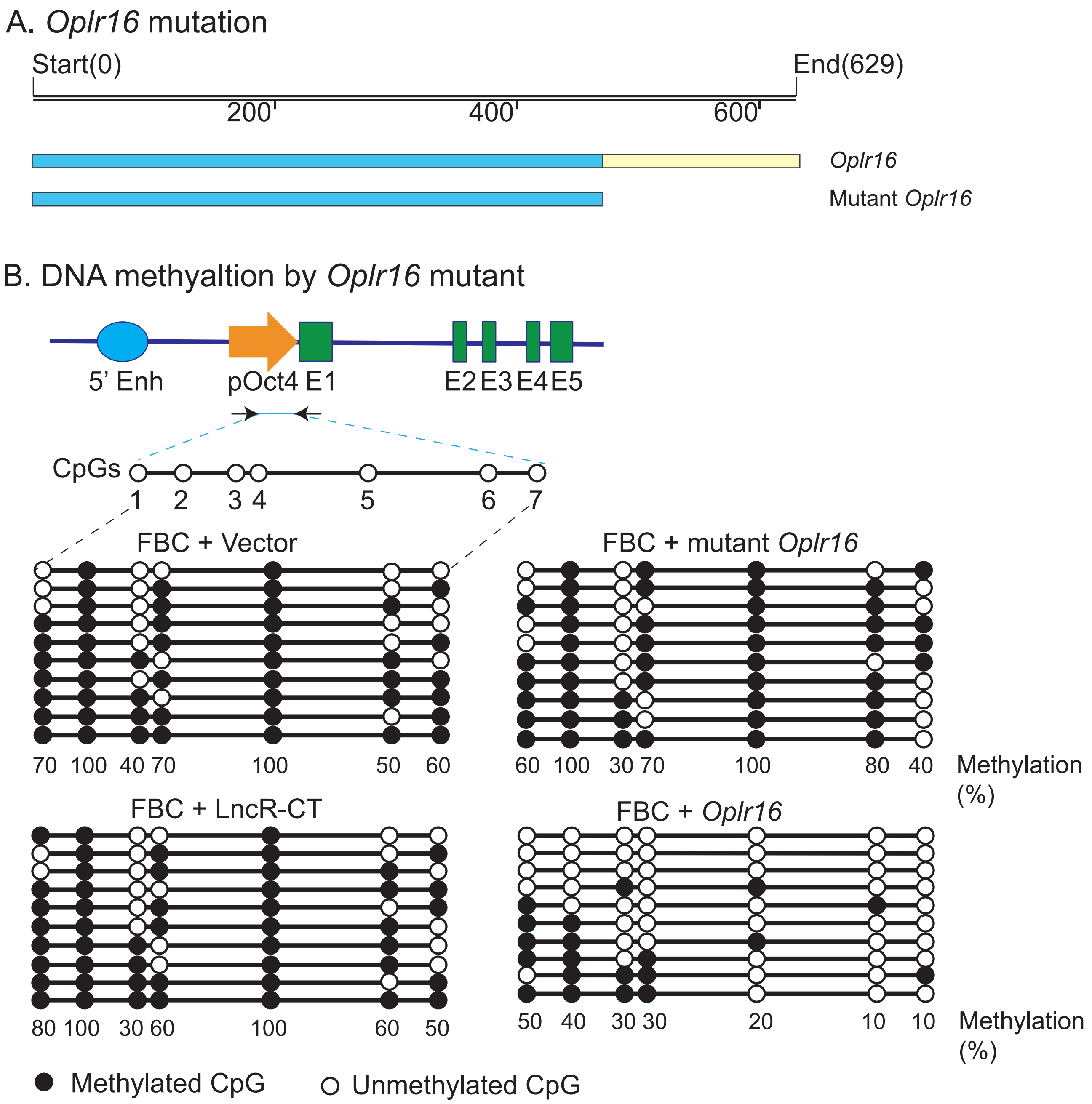

Figure S17. The 3'-*Oplr16* mutant loses the demethylation potential

A. Binding signals at the *Oct4* promoter

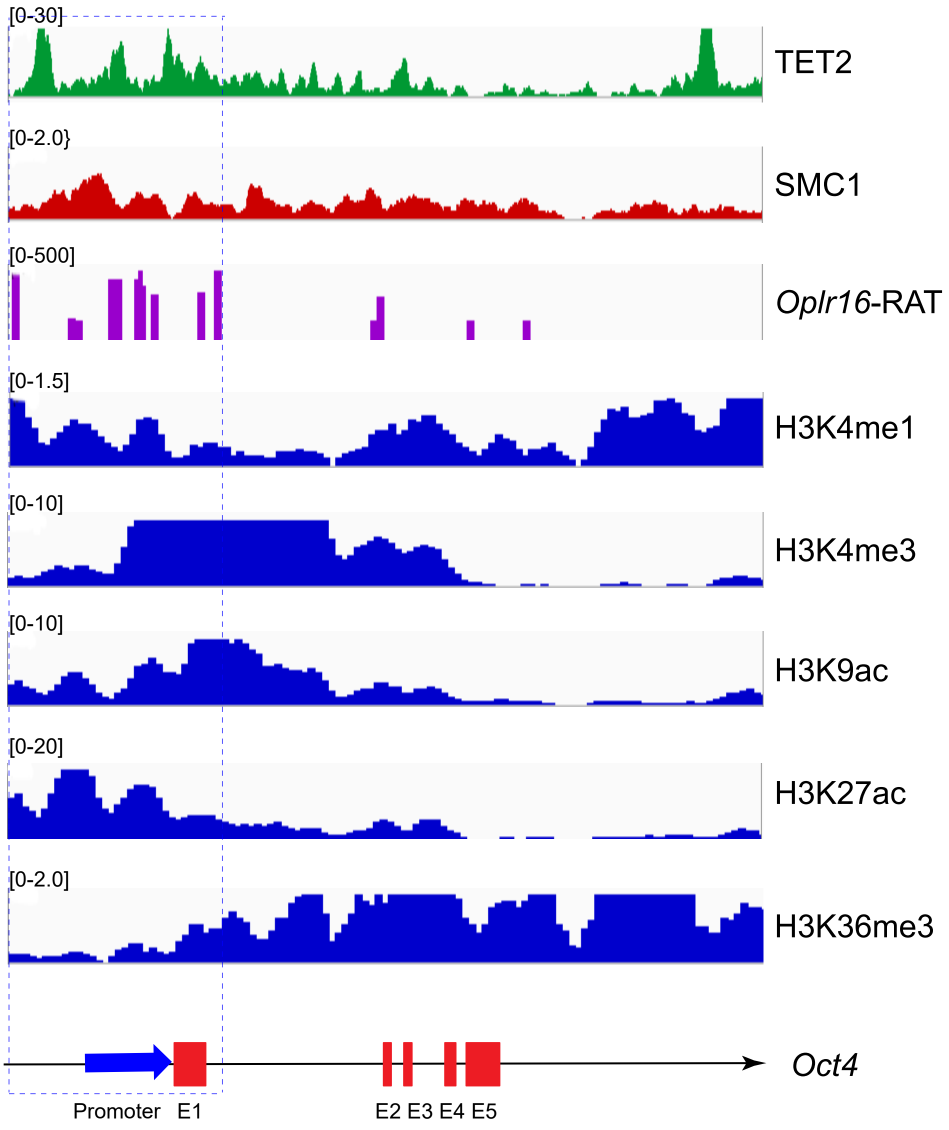

Figure S18. Binding signals and epigenotypes at the *Oct4* promoter
